# The lncRNA LAMP5-AS1 drives leukemia cell stemness by directly modulating DOT1L methyltransferase activity in *MLL* leukemia

**DOI:** 10.1186/s13045-020-00909-y

**Published:** 2020-06-17

**Authors:** Wen-Tao Wang, Tian-Qi Chen, Zhan-Cheng Zeng, Qi Pan, Wei Huang, Cai Han, Ke Fang, Lin-Yu Sun, Qian-Qian Yang, Dan Wang, Xue-Qun Luo, Yu-Meng Sun, Yue-Qin Chen

**Affiliations:** 1 MOE Key Laboratory of Gene Function and Regulation, State Key Laboratory for Biocontrol, School of Life Sciences, Sun Yat-sen University, Guangzhou, 510275 China; 2 Sun Yat-sen University Cancer Center, State Key Laboratory of Oncology in South China, Guangzhou, 510060 Guangdong China; 3 The First Affiliated Hospital of Sun Yat-sen University, Guangzhou, 510080 China

**Keywords:** lncRNA, LAMP5-AS1, DOT1L, H3K79 methylation, *MLL* leukemia, cell stemness

## Abstract

**Background:**

Mixed-lineage leukemia (*MLL*) gene rearrangements trigger aberrant epigenetic modification and gene expression in hematopoietic stem and progenitor cells, which generates one of the most aggressive subtypes of leukemia with an apex self-renewal. It remains a challenge to directly inhibit rearranged MLL itself because of its multiple fusion partners and the poorly annotated downstream genes of MLL fusion proteins; therefore, novel therapeutic targets are urgently needed.

**Methods:**

qRT-PCR, receiver operating characteristic (ROC), and leukemia-free survival analysis were used to validate LAMP5-AS1 (*LAMP5* antisense 1) expression and evaluate its clinical value. We performed in vitro and in vivo experiments to investigate the functional relevance of LAMP5-AS1 in *MLL* leukemia progression and leukemia cell stemness. RNA electrophoretic mobility shift assays (EMSA), histone methyltransferase assay, RNA pull-down assay, and RNA fluorescence in situ hybridization (FISH) were used to validate the relationship between LAMP5-AS1 and the methyltransferase activity of DOT1L. The downstream ectopic target genes of LAMP5-AS1/DOT1L were validated by the chromatin immunoprecipitation (ChIP) and western blot.

**Results:**

We discovered that a long noncoding RNA (lncRNA) LAMP5-AS1 can promote higher degrees of H3K79 methylation, followed by upregulated expression of the self-renewal genes in the *HOXA* cluster, which are responsible for leukemia stemness in context of *MLL* rearrangements. We found that LAMP5-AS1 is specifically overexpressed in *MLL* leukemia patients (*n* = 58) than that in the *MLL*-wt leukemia (*n* = 163) (*p* < 0.001), and the patients with a higher expression level of LAMP5-AS1 exhibited a reduced 5-year leukemia-free survival (*p* < 0.01). LAMP5-AS1 suppression significantly reduced colony formation and increased differentiation of primary *MLL* leukemia CD34+ cells. Mechanistically, LAMP5-AS1 facilitated the methyltransferase activity of DOT1L by directly binding its Lys-rich region of catalytic domain, thus promoting the global patterns of H3K79 dimethylation and trimethylation in cells. These observations supported that LAMP5-AS1 upregulated H3K79me2/me3 and the transcription of DOT1L ectopic target genes.

**Conclusions:**

This is the first study that a lncRNA regulates the self-renewal program and differentiation block in *MLL* leukemia cells by facilitating the methyltransferase activity of DOT1L and global H3K79 methylation, showing its potential as a therapeutic target for *MLL* leukemia.

## Background

*MLL* leukemia, which originates from a rearrangement of the mixed-lineage leukemia (*MLL*) gene by 11q23 translocation, is one of the most aggressive subtypes of acute leukemia with approximate 70% of infant leukemia patients and 7–10% of adult cases [1, 2]. Strong self-renewal capacity of this aggressive disease is generally deemed to be an important factor that results in limited treatment options and poor survival rates [3–7]. Recently, the *MLL* gene can recombine with more than 60 partners to acquire abnormal gain-of-function effects on aberrant epigenetic modification and proto-oncogene activation, as a result triggering dysregulated increased expression of the clustered homeobox A (*HOXA*) genes, which results in limitless self-renewal capacity necessary for leukemia initiation and propagation [3]. Hematopoietic malignancy caused by *MLL* translocation, especially *MLL-AF9*, has been reported to have an apex self-renewal to generate more distinct self-renewing progenitor-like leukemia cells that are resistant to chemotherapy and are responsible for relapse [6, 8–10]. Persistence of *MLL* leukemia clones after treatment including available cell cycle-based or signaling protein-targeted therapies is the critical factor for unsatisfactory treatment outcome [11, 12]. Thus, understanding the mechanism of how *MLL* leukemia cells to promote self-renewal and block differentiation could provide novel therapeutic strategies.

Ectopic and higher order H3K79 methylation, which triggers dysregulated activation of MLL fusion protein targets such as the *HOXA* genes, is believed to play an essential role in leukemogenesis and propagation of primary *MLL* leukemias [13, 14]. DOT1L is the only known histone methyltransferase that catalyzes histone 3 lysine 79 monomethylation (H3K79me1), dimethylation (H3K79me2), and trimethylation (H3K79me3), and higher degrees of H3K79 methylation have been indicated to be associated with elevated gene expression [15, 16]. Recent studies have demonstrated that the functions of DOT1L are regulated on multiple levels by the recruitment or blocking effector molecules, and misdirected localization and/or enhanced methyltransferase activity of DOT1L could lead to higher order H3K79 methylation and activation of MLL fusion protein target genes [14]. Thus, the identification and understanding of specific regulators of DOT1L in *MLL* leukemia could provide a precise target to block *MLL* leukemia cell stemness. However, little is known about the potential regulatory molecules that mediate the chromatin-modifying activity of DOT1L.

lncRNAs have been demonstrated to be key regulators in cellular metabolism processes, such as stemness maintenance [17–20], through diverse functions, including affecting chromatin epigenetic modification, protein complex constitution, and protein translation or degradation [18, 19, 21–23]. Remarkably, recent evidence indicates that lncRNAs could direct the development of hematopoiesis and leukemia. For instance, mouse lncRNA Spehd influences HSPC fate by participating in the oxidative phosphorylation pathway [24]. H19 lncRNA is pivotal for the development of embryonic hematopoietic stem cells [17]. LncRNA DANCR is upregulated in leukemia stem cells (LSCs), and its knockdown results in the decreased self-renewal of LSCs [25]. However, whether lncRNAs could specifically play regulatory roles in the balance between self-renewal and differentiation in *MLL* leukemia or serve as effector molecules of DOT1L to affect the abnormal activation of *HOXA* genes has not been reported yet.

We previously carried out a transcriptome microarray analysis of patient samples in the context of either *MLL* rearrangements or wild-type *MLL* (*MLL-*wt) and identified a distinct set of lncRNAs associated with *MLL* leukemia progression [26]. Specifically, we found that lncRNA LAMP5-AS1 showed the most significant difference between the two groups and prominently high expression in *MLL* leukemia patients. In this study, we reported that LAMP5-AS1 is necessary for cell self-renewal in *MLL* leukemia in view of its capacity to enhance the methyltransferase activity of DOT1L, which facilitates H3K79me2 and H3K79me3 modifications on the locus of the *HOXA* genes to upregulate their expression. LAMP5-AS1 knockdown remarkably inhibits the self-renewal capacity and promotes differentiation of *MLL* leukemia cells both in vivo and in vitro. We demonstrated that LAMP5-AS1 is crucial for the regulation of self-renewal in *MLL* leukemia and may be a valuable therapeutic target in this subtype of leukemia.

## Methods

### Leukemia patient samples

The clinical leukemia samples were obtained at the time of diagnosis or relapse and with informed consent from the first Affiliated Hospital of Sun Yat-sen University. Sample collection was approved by the Hospital’s Protection of Human Subjects Committee. The detail clinicopathological characteristics of the patients were summarized in Additional file 1: Table S1 and 2. The leukemia samples were stored in liquid nitrogen until used.

### Cell culture

Human *MLL* leukemia cells MOLM13, THP1, MV4-11, RS4-11, and HEK293T cells were purchased from American Type Culture Collection (ATCC, USA). MOLM13, THP1, and RS4-11 were cultured in RPMI-1640 medium (HyClone, USA); MV4-11 cells were cultured in IMDM (HyClone), and HEK 293T (ATCC) were cultured in DMEM (Gibco) supplemented with 10% FBS (HyClone) at 37 °C in a 5% CO_2_ atmosphere. The primary cells were from the patients with *MLL* leukemia and cultured in IMDM (HyClone) supplemented with 20% FBS [27, 28]. Primary CD34+ blasts cells sorted from *MLL* leukemia patients were either cultured in IMDM medium supplemented with 20% FBS and 10 ng/mL of SCF, TPO, Flt-3 L, IL-3, and IL-6 [29].

### Xenotransplantation experiments

Five-week-old NOD-SCID mice were maintained under specific pathogen-free conditions in the Laboratory Animal Center of Sun Yat-sen University. All experiments on animals were performed according to the institutional ethical guidelines for animal experiments. MOLM13 cells transduced with control or knockdown lentivirus (GFP+ cell populations) were tail vein injected into the mice (5 × 10^6^ cells in 150 μL PBS per mouse). Three weeks after inoculation, xenografted mice were sacrificed for analysis. Human cell engraftment (GFP+ cell populations) in the bone marrow, peripheral blood, liver, and spleen was evaluated by flow cytometry or hematoxylin and eosin (H&E) staining performed as described. The remaining mice were performed the survival assay.

### RNA electrophoretic mobility shift assays (EMSA)

The biotin-labeled RNA probes of variant LAMP5-AS1 truncated mutants were generated using the TranscriptAid T7 High Yield Transcription Kit (Thermo Fisher, USA) according to the manufacturer’ s guidelines. Recombinant DOT1L (residues 1-416aa) protein was mixed with variant RNA probes in the EMSA binding buffer (10 mM HEPES, pH 7.3, 20 mM KCl, 1 mM MgCl_2_, 1 mM DTT, 5% glycerol, 100 μg/mL transfer RNA). The reaction systems were incubated at room temperature for 30 min. Then, the complexes were separated using non-denaturing polyacrylamide gel electrophoresis. Biotinylated RNA was measured in the blots with a chemiluminescent EMSA Kit (Beyotime, China).

### Histone methyltransferase assay

Histone methyltransferase assay was performed as previously described [30]. Oligonucleosomes purified from HeLa cells were purchased from Millipore. Briefly, recombinant DOT1L (residues 1-416aa) were incubated with oligonucleosomes in the reaction buffer (50 mM NaCl, 50 mM Tris-HCl, pH 8.5, 5 mM MgCl_2_, 1 mM DTT, and 10 μM SAM) at 30 °C for 1 h. Additional LAMP5-AS1 with different concentration was added to the reactions. The reaction systems were supplemented with 1× SDS loading buffer and were boiled at 100 °C for 10 min. The proteins were separated in a 12% SDS-PAGE gel, and H3K79me2 and H3K79me3 were assessed by immunoblotting. H3 was served as a control.

### Culture and transduction of primary leukemia cells

Cells were thawed in a 37 °C water bath, washed once with Iscove’s modified Dulbecco medium (IMDM) containing 20% fetal bovine serum (FBS). Then, the cells were culture in IMDM supplemented with 20% FBS. CD34+ leukemia stem/progenitor cells were purified using human CD34 MicroBead Kit (Miltenyi Biotec, Auburn, CA) and fluorescence-activated cell sorting (FACS). The transient transfections of primary leukemia cells were performed using the Neon Transfection System with 10 μL reactions according to the manufacturer’s guidelines (Invitrogen, USA).

### Plasmid construction

The wild-type DOT1L-CDS-full length and DOT1L truncated mutants (1-416aa, 417-822aa, 1-822aa, and 823-1537aa) were PCR-amplified from THP1 cDNA using the primers shown in Additional file 1: Table S3. Then, the PCR product was purified and cloned into pCDH-CMV-MCS-EF1-Puro eukaryotic expression vector (pCDH-DOT1L) which has be reconstructed with a FLAG-tag fusion at the N terminal and then derived a FLAG-DOT1L and truncated mutants fusion protein once they expressed. Similarly, the DOT1L (1-416aa) truncated mutants (1-416aa, 1-390aa, 1-407aa, 360-416aa, 1-416aa deletion 390-407) were also PCR-amplified from THP1 cDNA using the primers shown in Additional file 1: Table S3, and then were purified and cloned into pcDNA3.1 eukaryotic expression vector which has been reconstructed with a HA-tag fusion at the N terminal and then derived a HA-DOT1L truncated mutant fusion protein once they expressed.

For purification of 1-416aa DOT1L in vitro, corresponding cDNA fragment of the 1-416aa DOT1L was amplified by PCR and then the cDNA fragment was cloned into a prokaryotic expression vector pET-N-GST-Thrombin-C-His that contains an N-terminal fusion of a GST tag.

For stable LAMP5-AS1 knockdown vectors, the DNA oligos encoding shRNAs targeting LAMP5-AS1 (sh-LAMP5-AS1-1 and sh-LAMP5-AS1-2) were synthesized and cloned into the pGreenPuro™ eukaryotic expression vector, with sh-NC as the negative control (Additional file 1: Table S4).

### Lentiviral preparation and infection

Lentivirus carrying shRNAs was made in the 60-mm culture dish by transfecting packaging cell HEK293T with Lentivector Expression Systems (System Biosciences, Germany) consisting of pPACKH1-GAG, pPACKH1-REV, and pVSV-G. Virus was harvested 48 and 72 h after transfection. Lentivirus Precipitation Solution (System Biosciences, Germany) was used to precipitate the virus.

For stable expression assays, 3 × 10^5^ MOLM13, THP1, and MV4-11 were prepared for each infection system. The cells were centrifuged and resuspended in 300 μL virus suspension, followed by incubation at 37 °C and 5% CO_2_ for 24 h. Then, the cells were centrifuged, washed, and resuspended in fresh medium containing 1 μg/ml puromycin and 1% penicillin-streptomycin. To confirm target knockdown, cells were collected for qRT-PCR analysis.

### Transfection of cell lines

SiRNAs were transfected into MOLM13, THP1, MV4-11, and RS4-11 cells at a final concentration of 50 nM with Neon™ Transfection System 10 μL Kit using the Neon Transfection System (Invitrogen, USA). HEK293T cells were transfected using the Lipofectamine 2000/3000 (Invitrogen, USA). Cells were collected 48 or 72 h after transfection. The siRNA sequences were listed in Additional file 1: Table S4.

### RNA isolation and quantitative real-time PCR (RT-PCR)

Total RNA was extracted from bone marrow and cell samples using an Invitrogen™ TRIzol™ Kit (Thermo Fisher, USA) according to the manufacturer’s instructions. All RNA samples were stored at − 80 °C before reverse transcription and quantitative RT-PCR. RNA was reverse-transcribed into cDNA with the PrimeScript® RT reagent Kit with gDNA Eraser (Takara, Japan). Quantitative RT-PCR for lncRNA and mRNA was performed using the SYBR Premix ExTaq real-time PCR Kit (Takara, Japan) according to the manufacturer’s instructions. All of the data were normalized to GAPDH expression as a control. The expression level for each lncRNA and mRNA was determined using the 2^−△△Ct^ method. All primers were confirmed by sequencing the PCR product fragments, as shown in Additional file 1: Table S3.

### Cell nucleus/cytoplasm fraction isolation

Cell nuclear and cytoplasmic fractions were isolated from cell samples using the NE-PER Nuclear and Cytoplasmic Extraction Reagents (Thermo Fisher, USA) according to the manufacturer’s instructions.

### Protein extraction

Total protein was extracted from cells using RIPA lysis buffer (Beyotime Biotechnology, China) with 1× complete ULTRA (Roche, USA); protein was extracted from bone marrow samples using an Invitrogen™ TRIzol™ Kit (Thermo Fisher, USA) with the Thermo Scientific™ Halt™ Protease Inhibitor Cocktail (Thermo Fisher, USA) according to the manufacturer’s instructions.

### Colony forming cell (CFC) assay

A total of 10,000 *MLL* leukemia patient cells or CD34+ *MLL* leukemia cells were used per CFC assay and were added to 3 mL of methylcellulose media (R&D, USA). The vial was vigorously vortexed to thoroughly mix cells with the media. The media was allowed to stand until air bubbles disappeared. Next, we added 1.1 mL of the final cell mixture to each 35-mm culture dish. Two sample dishes and an uncovered dish containing 3–4 mL of sterile water were then placed into a 100-mm culture dish with a cover. Cells were incubated at 37 °C and 5% CO_2_, and the total colonies were counted after 7 days post plating.

Colony assay for *MLL* leukemia cell lines MOLM13 and THP1 was performed by plating 5000 cells on methylcellulose media (R&D, USA). Cells were incubated for 14 days respectively at 37 °C and 5% CO_2._

### Cell differentiation and morphological assay

For flow cytometric analysis, primary *MLL* leukemia patient cells, CD34+ *MLL* leukemia cells, and MOLM13, THP1, and RS4-11 with different treatments were harvested and washed with cooling PBS and were stained with the specific antibodies of differentiation markers (anti-CD14 and anti-CD11b for the differentiation of *MLL* myeloid leukemia cells, anti-CD19 for the differentiation of *MLL* lymphoid leukemia cells, BD Biosciences) with 0.5% FBS. The cells were incubated at 37 °C in the dark for 30 min and then were analyzed on a BD FACSCalibur analyzer (BD Biosciences, USA).

For morphological analysis, 50,000 cells of MOLM13 or THP1 were collected at 1000 rpm for 5 min. Slides were let dry and stained with Wright-Giemsa Stain Solution (Sangon biotech, China).

### Limiting dilution assay

MOLM13 cells transduced with control or LAMP5-AS1 knockdown lentivirus were tail vein injected into 5-week-old NOD-SCID mice with four (10,0000, 50,000, 10,000, 5000) different doses of cells for each group of 4 mice. The number of recipient mice developed leukemia within 4 weeks after inoculation, and a recipient mouse was considered positive if GFP+ cells constituted more than 1.0% of all nucleated cells in the blood. Limiting dilution analysis (ELDA) online software (http://bioinf.wehi.edu.au/software/elda/) [31] was used to estimate the frequency of leukemia stem/progenitor-like cells in MOLM13 upon different treatments in vivo.

### 5′ RACE and 3′ RACE

Total RNA from THP1 cells was extracted using Trizol reagent (Invitrogen, CA, USA) according to the manufacturer’ s guidelines. The 5′- and 3′-ends of cDNA were acquired using a 5′-FULL RACE Kit with TAP (Takara, Japan) and 3′-FULL RACE Core Set with PrimeScript RTase (Takara, Japan) respectively according to the manufacturer’s instructions. PCR products were obtained and then cloned into pEASY-T3 (TransGen Biotech, China) for further sequencing. All of the primers for RACE experiments are listed in Additional file 1: Table S3.

### RNA pull-down assay

We performed pull-down assays with biotinylated LAMP5-AS1 using a Pierce™ Magnetic RNA-Protein Pull-Down Kit (Thermo Fisher, USA) according to the manufacturer’s instructions. First, the LAMP5-AS1 and LAMP5-AS1 antisense sequences (primers are listed in Additional file 1: Table S3) were reverse-transcribed in vitro by PCR with T7 RNA polymerase using the TranscriptAid T7 High Yield Transcription Kit (Thermo Fisher, USA) with biotin-labeled UTP mix, and then the RNAs were purified using the Thermo GeneJET RNA Purification Kit (Thermo Fisher, USA). After folding, biotin-labeled RNAs were mixed with THP1 cell extract (containing 2 mg total protein) in 400 μL RIP buffer and incubated at RT (room temperature) for 1 h. Next, 50 μL of washed streptavidin magnetic beads was added to each reaction and further incubated at RT for another hour. Beads were washed briefly with RIP buffer six times and then boiled in SDS loading buffer. Finally, the enriched proteins were resolved via SDS-PAGE and silver stained followed by mass spectrometry (MS) identification (FitGene Biotechnology, China) and western blotting.

For LAMP5-AS1 truncated mutant pull-down assays, we performed the tRSA RNA pull-down system based on previous publications with modifications. LAMP5-AS1 full-length or truncated mutant sequences were first cloned into pcDNA3.1 plasmid containing the 5′ terminal tRSA tag. The plasmids were used as templates to in vitro transcribe RNA products using the TranscriptAid T7 High Yield Transcription Kit (Thermo Fisher, USA). Then, the RNA products were purified using the GeneJET RNA Purification Kit (Thermo Fisher, USA). The following procedures were based on the Pierce™ Magnetic RNA-Protein Pull-Down Kit (Thermo Fisher, USA).

### RNA fluorescence in situ hybridization (FISH) and immunofluorescence microscopy

To detect the subcellular location of LAMP5-AS1 RNA, we carried out FISH in THP1 and MV4-11 cells using the Ribo^TM^ Fluorescent In Situ Hybridization Kit (RiboBio, China). Cells were washed briefly with PBS and then fixed in 4% formaldehyde for 15 min at room temperature. Cells were permeabilized in PBS containing 0.5% Triton X-100 on ice for 5 min and then blocked in the preliminary hybridization solution for 30 min at room temperature after three washes with PBS for 10 min each. Hybridization was carried out using the LAMP5-AS1 FISH Probe Mix (RiboBio, China) in a humidified chamber at 37 °C for 12–16 h. Cells were rinsed with SSC buffer in accordance with the order 4×, 2×, and 1×. For co-localization studies, after RNA FISH, cells were fixed again for 5 min in 2% formaldehyde and subjected to immunofluorescence with anti-DOT1L primary antibody and fluorescent secondary antibody were sequentially (antibodies were listed in Additional file 1: Table S5). The nuclei were counterstained with DAPI. Cells were observed on a Zeiss7 DUO NLO confocal laser microscope (Carl Zeiss, Germany).

### Purification of DOT1L (1-416aa)

The construction of *GST*-1-416aa DOT1L expression vector was described above in Plasmid Construction. Recombinant DOT1L (residues 1-416aa) with a termination codon in pET-N-GST-Thrombin-C-His was transformed into *E*. *coli* expression strain BL21 [Transetta(DE3) chemically competent cell (Transgen biotech, CD801)] for expression of GST-DOT1L (1-416aa). Five milliliters of Luria-Bertani (LB) culture supplemented with kanamycin was inoculated with a single colony at 37 °C. After overnight growth, the culture was diluted 100-fold into 300 mL LB supplemented with kanamycin. Protein expression was induced in the presence of 0.4 mM IPTG at 16 °C overnight. Then, the cell pellets were collected by centrifugation at 5000 rpm, 4 °C for 10 min, and were suspended in lysis buffer (500 mM NaCl, 50 mM Tris pH 8.0, 5% Glycerol, 0.5 mM (DTT), and 1x Protease Inhibitor (cOmplete EDTA free, Roche)). The cells were lysed on ice for 30 min, followed by sonication on ice with 4 s on/6 s off for 25 min. After centrifugation, the supernatant was applied to a ProteinIso® GST Resin column (TransGen Biotech, China) and the column was washed 3 times using lysis buffer. GST-DOT1L (1-416aa) protein was digested with Thrombin (sigma, USA) in digestion buffer (75 mM NaCl, 20 mM Tris pH 8.0, 5% Glycerol, 1 mM DTT) overnight at 4 °C to cleave off GST-tag. Finally, the tag-free DOT1L (residue 1-416aa) was pooled and the buffer exchanged into storage buffer (200 mM NaCl, 30 mM Tris pH 8.0,1 mM Tris(2-carboxyethyl)phosphine (TCEP), 20% glycerol) using an Amicon Ultra spin concentrator (Millipore, USA). The pure concentrated protein at 25 μM was then stored at − 80 °C until use.

### Chromatin immunoprecipitation (ChIP) and ChIP-seq

ChIP analyses were performed on chromatin extracts from THP1 and MOM13 cells using a Magna ChIP™ G - Chromatin Immunoprecipitation Kit (17-611) (Merck Millipore, Germany) with di- and tri-Histone H3 (Lys79) according to the manufacturer’s standard protocol. In this assay, samples incubated with Rabbit IgG served as the negative control. The fold enrichment of H3K79me2/3 was quantified by quantitative RT-PCR and calculated relative to the input chromatin. The primers used for ChIP-qPCR analysis were listed in Additional file 1: Table S3. Eluted DNA fragments were also subjected to sequencing using the next-generation Illumina sequencing. The ChIP-seq data are uploading to the gene expression omnibus (GEO) database and the number is GSE150483.

### Immunoblotting and RNA immunoprecipitation (RIP)

Proteins extracted from primary patient cells or cell lines were resolved by 7.5%, 10%, or 12% Bis-Tris polyacrylamide gels and were transferred to polyvinylidene fluoride membranes. Membranes were blocked in 5% BSA for 1 h and probed with the appropriate antibody overnight at 4 °C and then were incubated with horseradish peroxidase-conjugated secondary antibodies at room temperature for 1 h. Membranes were visualized with an enhanced chemiluminescence detection system. Detail information of antibody was mentioned in Additional file 1: Table S5.

For RIP assays, FLAG- or HA-tagged fusion proteins were used. In the RIP experiment with FLAG-DOT1L (N- terminal) or HA-DOT1L (N- terminal) truncated mutants in 293T cells, anti-FLAG (Sigma, USA) or anti-HA (Sigma, USA) was used along with an EZ-Magna RIP™ RNA-Binding Protein Immunoprecipitation Kit (17-701) (Merck Millipore, Germany) according to the manufacturer’s instructions. All proteins for RIP were lysed with cell lysis buffer supplemented with Thermo Scientific™ Halt™ Protease Inhibitor Cocktail (Thermo Fisher, USA). Finally, all samples were suspended in 5× loading buffer and then denatured for 5 min at 100 °C, separated via SDS-PAGE, transferred to PVDF membranes, and blotted.

### Statistical analysis

Pearson’s correlation coefficient was used to determine the correlation between lncRNA and MLL fusion protein levels. Mann-Whitney test was used to analyze the LAMP5-AS1 levels between patients with or without MLL fusion proteins. Fisher’s exact test was used to determine the significance of differentially expressed lncRNA and mRNA levels between the two groups. Data are expressed as the mean ± SEM of three independent experiments. One-way ANOVA was performed to compare multiple groups, and the LSD *t* test was used to analyze multiple comparisons. The probability of leukemia-free survival at 5 years was the study end-point. Leukemia-free survival was calculated from the date of complete remission (CR) until either relapse or death in remission. Leukemia-free survival was analyzed using the Kaplan-Meier method with a log-rank test. Two-tailed tests were used for univariate comparisons. For univariate and multivariate analysis of prognostic factors, a Cox proportional hazard regression model was used. *p* < 0.05 was considered statistically significant

## Results

### LAMP5-AS1 is required to sustain self-renewal capacity against *MLL* leukemic cell differentiation

**Fig. 1:**
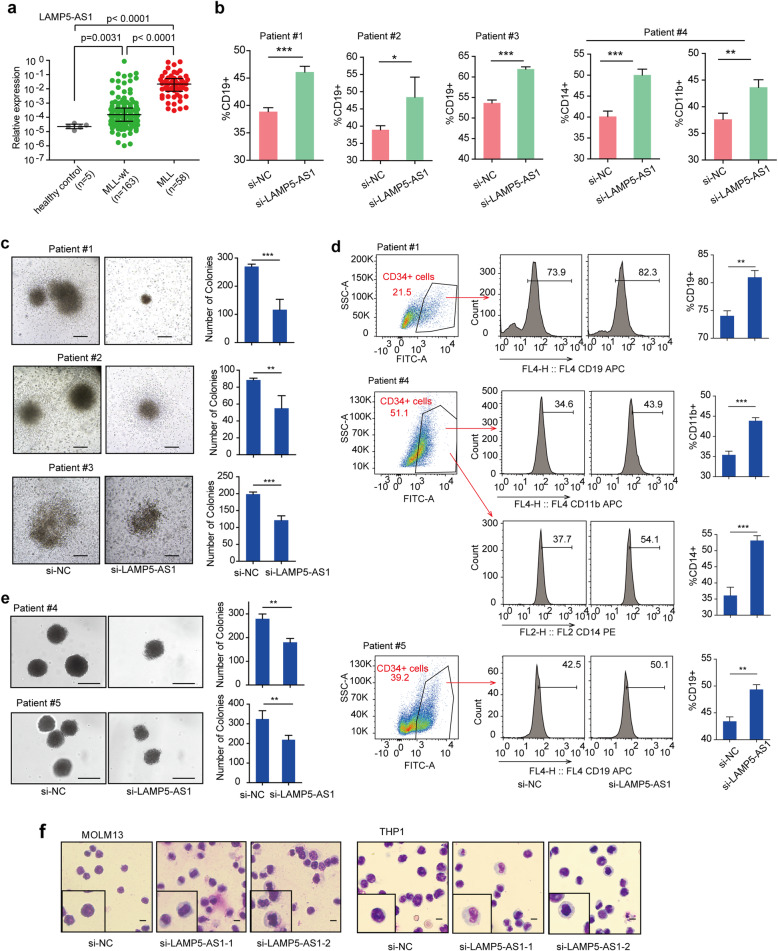
Impact of aberrantly expressed LAMP5-AS1 on primary *MLL* leukemia self-renewal and differentiation. **a** LAMP5-AS1 showed different expression levels in *MLL* leukemia (*n* = 58) and *MLL-*wt leukemia (*n* = 163) patients and healthy controls (*n* = 5) (*p* < 0.05 between each two groups). *MLL* leukemia groups have the highest expression level of LAMP5-AS1. **b**, **d** Flow cytometric analysis of the CD19+, CD11b+, or CD14+ cell populations in primary *MLL* leukemia cells (**b**) and CD34+ *MLL* leukemia cells (**d**) after transduction with LAMP5-AS1 siRNAs or control. Error bars reflect ± SEM (*p < 0.05, **p < 0.01, ***p < 0.001). **c**, **e** Morphology of colonies of primary *MLL* leukemia cells (**c**) and CD34+ *MLL* leukemia cells (**e**) upon siRNA-mediated knockdown of LAMP5-AS1. Scale bars, 100 μm. Error bars reflect ± SEM (*p < 0.05, **p < 0.01, ***p < 0.001). **f** Wright-Giemsa staining of MOLM13 and THP1 cells after transduction with LAMP5-AS1 siRNAs or control. Scale bar, 20 μm.

LAMP5-AS1 is a 1928 nt lncRNA originating from the gene that is located on the antisense strand of the coding gene lysosome-associated membrane protein 5 (*LAMP5*) on chromosome 20 [32, 33]. Two isoforms of the lncRNA were found when using rapid amplification of cDNA ends (RACE) to verify both the 5′ and 3′ terminal sequences of LAMP5-AS1 (Additional file 1: Figure S1a). We further investigated the expression levels of the two LAMP5-AS1 isoforms in *MLL* leukemic cell lines, and only one transcript (transcript 1) showed a high expression level (Additional file 1: Figure S1b). Thus, we mainly focused on LAMP5-AS1 transcript 1 in the following studies. To reveal the clinical relevance of LAMP5-AS1, we analyzed its expression pattern using a large cohort of clinical samples with and without *MLL* rearrangements; a significantly higher expression level of LAMP5-AS1 was found in the *MLL* leukemia patient group than in the *MLL*-wt leukemia or healthy control sets (*p* < 0.001) (Fig. 1a; Additional file 1: Table S1). The higher expression level of LAMP5-AS1 was also found in all leukemic cell lines with different *MLL* translocations (Additional file 1: Figure S2a). The specifically higher expression level of LAMP5-AS1 in *MLL* leukemia prompted us to explore whether the lncRNA is essential in *MLL* leukemia.

It has been reported that hematopoietic malignancy caused by *MLL* translocation has an apex self-renewal to generate more distinct self-renewing progenitor-like leukemia cells [6, 8]. Therefore, we firstly investigated whether LAMP5-AS1 affects self-renewal and differentiation in leukemia cells. Primary cells from four *MLL* leukemia patient bone marrows were used. Additional file 1: Table S2 showed the clinicopathologic features of primary *MLL* leukemia patient samples, including three acute lymphoid leukemia patients (one with *MLL-AF9*, another one with *MLL-AF4*, and the third one with *MLL* fused by non-classical partner) and one acute myeloid leukemia patient (*MLL*-*AF10*). Upon LAMP5-AS1 knockdown (Additional file 1:Figure S2b), the primary cells were noticeably induced toward lymphocyte or monocyte/macrophage differentiation with significant increases in the specific lymphoid differentiation marker CD19 or the myeloid differentiation marker CD14 and CD11b, respectively (Fig. 1b; Additional file 1: Figure S2c). We further found that LAMP5-AS1 suppression significantly impaired the capacity of primary cells from *MLL* leukemia patients to form colonies in methylcellulose, which was in parallel with the reduced proportion of CD34^+^ leukemia cells (Fig. 1c; Additional file 1: Figure S2d). When we further isolated primary CD34^+^ cells from *MLL* leukemia patient cells and inhibited their LAMP5-AS1 expression, we found that LAMP5-AS1 knockdown promoted the level of differentiation in leukemia stem and progenitor cells (Fig. 1d). Notably, *MLL* leukemia CD34^+^ cells showed reduced colonies in the LAMP5-AS1 knockdown groups than in the control group, demonstrating impaired proliferation and self-renewal potential of *MLL* leukemia CD34^+^ cells when LAMP5-AS1 was depleted (Fig. 1e). Additionally, the regulation of LAMP5-AS1 on self-renewal and differentiation was further verified in the *MLL* leukemia cell lines MOLM13, THP1, MV4-11, and RS4-11. LAMP5-AS1 suppression (Additional file 1: Figure S3a and b) led to a drastic increase in differentiation assessed by the frequency and fluorescence intensity of the myeloid markers CD11b and CD14 or the lymphoid markers CD19 (Additional file 1: Figure S3c-e) and cellular morphology (Fig. 1f). Similarly, LAMP5-AS1 knockdown prominently inhibited colony formation in both THP1 and MOLM13 cells in vitro (Additional file 1: Figure S3f). Together, these results demonstrated that LAMP5-AS1 knockdown substantially inhibits self-renewal capacity and promotes differentiation of *MLL* leukemia cells, implying that LAMP5-AS1 plays a role in the progression of *MLL* leukemia.

### LAMP5-AS1 facilitates *MLL*-driven leukemia progression in vivo

**Fig. 2:**
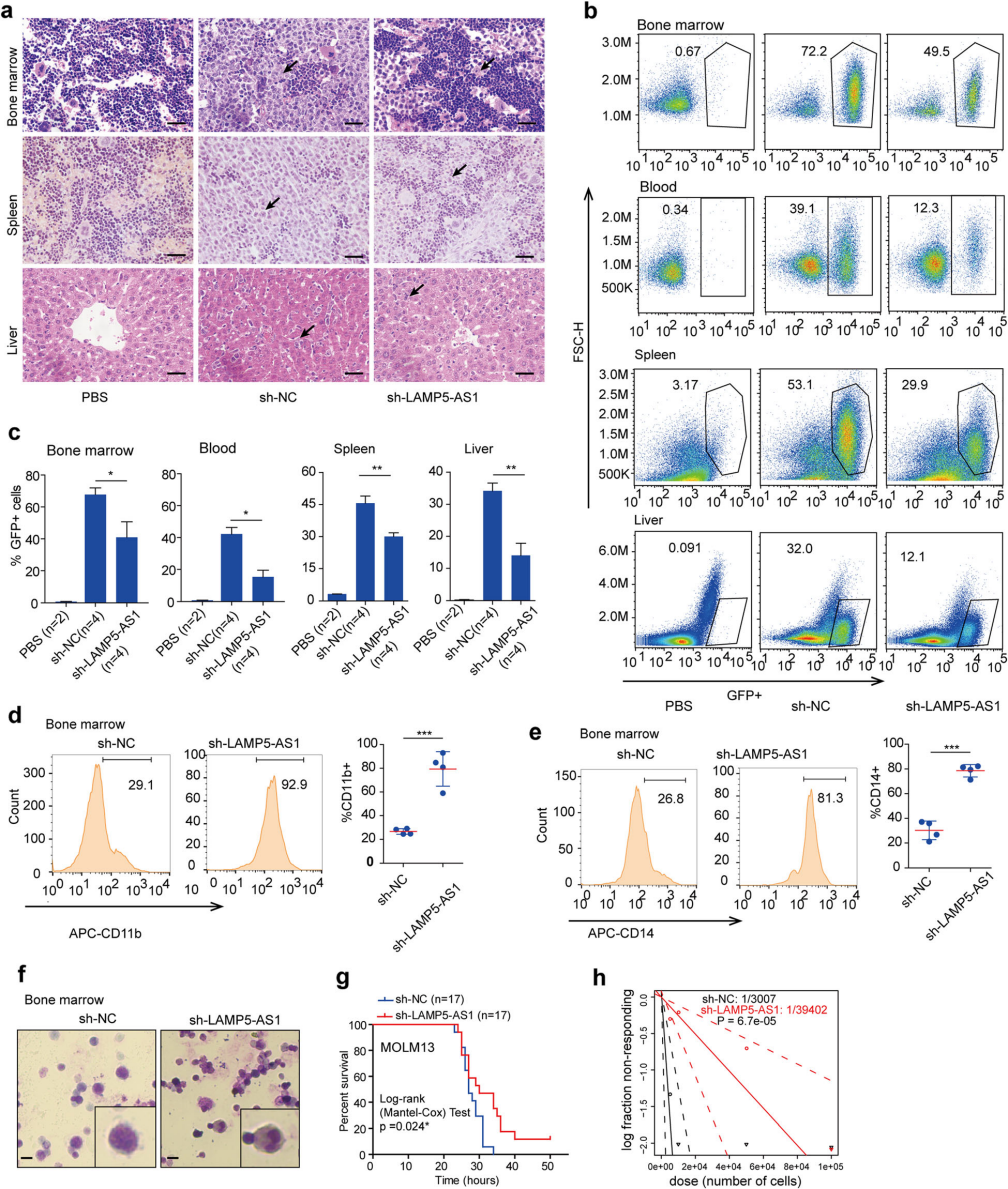
LAMP5-AS1 knockdown impairs the progression and infiltration of *MLL* leukemia in vivo*.***a** H&E staining in bone marrow, spleen, and liver sections from mice engrafted with LAMP5-AS1 knockdown MOLM13 cells compared with that in control mice. Mice treated with PBS were the negative controls. GFP+ cells in the tissues are indicated by black arrows. A representative image from three independent mice is shown. Scale bars, 50 μm. **b** Representative flow cytometry graphs showing the decreased levels of blasts in organ samples from mice treated with LAMP5-AS1 knockdown MOLM13 cells relative to the levels in control mice. Mice treated with PBS were used as the negative controls. **c** Histogram plots show the statistical values for **b**. Error bars reflect ± SEM (*p < 0.05, **p < 0.01). **d**, **e** Representative flow cytometry graphs showing the CD11b (**d**) and CD14 (**e**) markers in the bone marrow from mice treated with LAMP5-AS1 knockdown MOLM13 cells relative to the levels in control mice. The values analyzed by error bars reflect ± SEM (*p < 0.05, **p < 0.01). **f** Wright-Giemsa staining of bone marrow smears from mice engrafted with human MOLM13 cells transfected with sh-NC or sh-LAMP5-AS1. The staining was enlarged under a × 40 objective lens. Scale bars, 20 μm. **g** Kaplan-Meier survival curves showing the survival of mice implanted with sh-NC- or sh-LAMP5-AS1-transfected MOLM13 cells. *p* values were calculated using a log-rank (Mantel-Cox) test (*p < 0.05). **h** Limiting dilution assays using LAMP5-AS1 knockdown and control MOLM13 cells. The estimated the frequency of leukemia cells with self-renewal capacity is shown on the plot. Mice developed leukemia within 4 weeks post injection, and a recipient mouse was considered positive if GFP+ cells constituted more than 1.0% of all nucleated cells in the blood

To further investigate the role of LAMP5-AS1 in *MLL* leukemogenesis in vivo, we generated NOD/SCID mouse xenograft models with MOLM13 cells. The xenograft model was established by injecting MOLM13 cells transfected with LAMP5-AS1 short hairpin RNA (named sh-LAMP5-AS1) and control (named sh-NC) into the tail vein of NOD/SCID mice. We killed the mice after 3 weeks and then assessed the organ infiltration regulated by LAMP5-AS1 knockdown via hematoxylin and eosin (H&E) staining and flow cytometry. H&E staining results showed that the amounts of *MLL* leukemia cells in the bone marrow, spleen, and liver from sh-LAMP5-AS1-transfected mice were prominently reduced compared with those from sh-NC–transfected mice (Fig. 2a), indicating that LAMP5-AS1 suppression impaired *MLL* leukemia cell infiltration. Consistent results were also shown that the organ infiltration capacity of LAMP5-AS1-depleted *MLL* leukemia cells was significantly decreased as demonstrated by the declined percentages of GFP+ cell populations in the bone marrow, blood, spleen, and liver from sh-LAMP5-AS1-transfected mice (Fig. 2b, c). Dramatic increases in differentiation markers CD11b and CD14 (Fig. 2d, e) and prominently lobulated karyotypes were observed in GFP+ cell population from sh-LAMP5-AS1-transfected mice (Fig. 2f), implying that LAMP5-AS1 is necessary to inhibit differentiation in *MLL* leukemia cells. Notably, the mice in the sh-LAMP5-AS1 groups survived longer than those in the sh-NC groups (Fig. 2g), suggesting that LAMP5-AS1 knockdown could inhibit *MLL* leukemia progression.

Then, we further used a limiting dilution assay of MOLM13 cells transfected with sh-LAMP5-AS1 or sh-NC in mice to investigate the frequency of *MLL* cells with stemness characteristics when LAMP5-AS1 was suppressed. LAMP5-AS1 suppression greatly reduced the engraftment of GFP+ cell populations and the frequency of MOLM13 cells with self-renewal capacity in mice at 4 weeks after transplantation (Fig. 2h). Overall, we found that LAMP5-AS1, a specifically highly expressed lncRNA in *MLL* leukemia, was necessary for certain leukemia to promote self-renewal capacity and inhibit differentiation.

### LAMP5-AS1 directly binds to H3K79 methyltransferase DOT1L at its Lys-rich region of catalytic domain

**Fig. 3:**
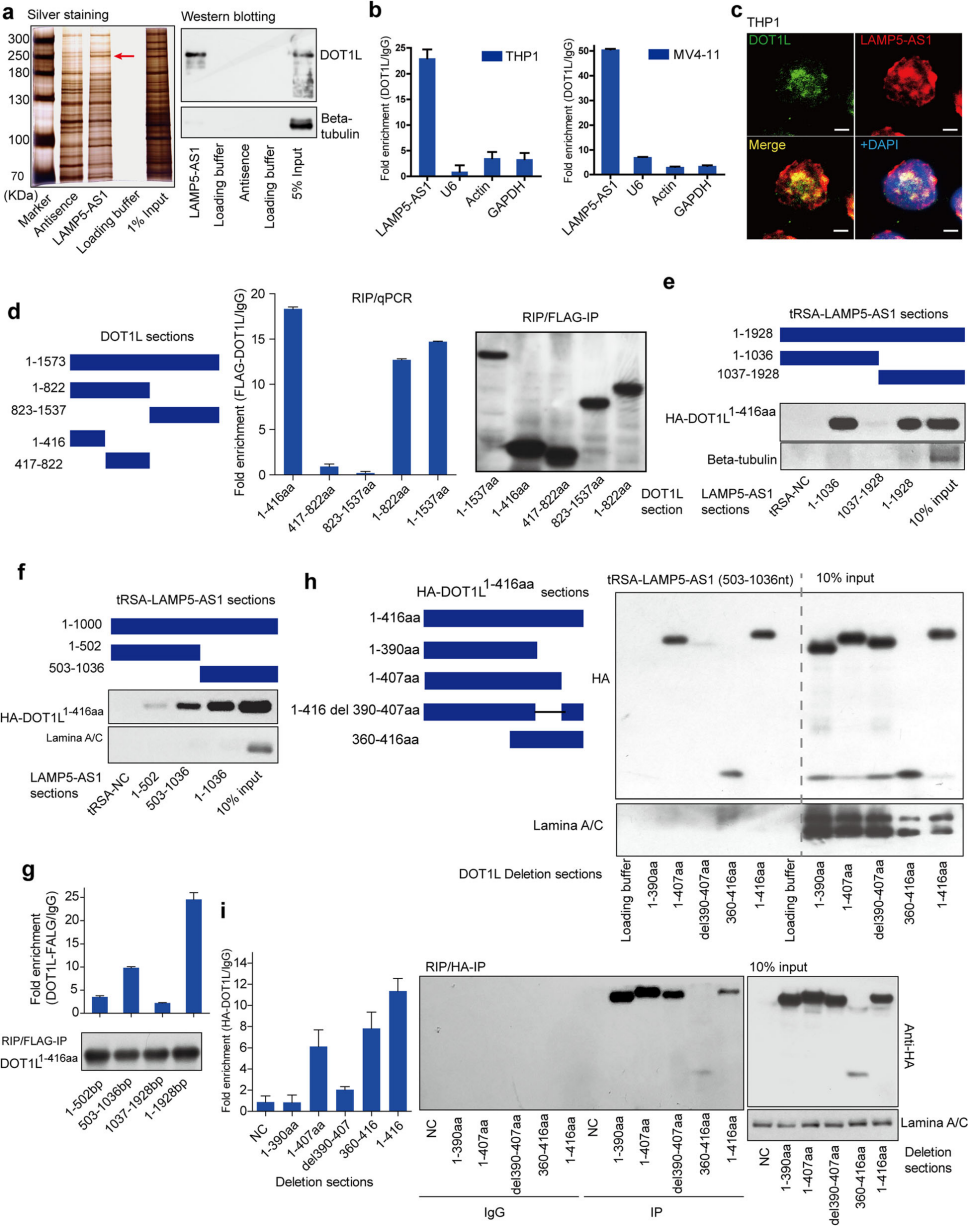
Identification and characterization of LAMP5-AS1 binding to DOT1L. **a** Western blotting and silver staining of SDS-PAGE followed by mass spectrometry showed that DOT1L was enriched by LAMP5-AS1 pull-down assays. Beta-tubulin as the negative control. **b** LAMP5-AS1 was significantly enriched by the RIP of DOT1L in *MLL* leukemia cells. Error bars reflect ± SEM from three independent experiments. **c** RNA FISH and IF experiments showed that LAMP5-AS1 co-localized with DOT1L in the cell nucleus. Scale bar, 5 μm. **d** RIP-qPCR for the LAMP5-AS1 and FLAG-tagged DOT1L sections. DOT1L (1-416 aa, 1-822 aa, and 1-1537 aa) presented significantly higher enrichment of LAMP5-AS1. Error bars reflect ± SEM from three independent experiments. Immunoblotting indicated the successful RIP assay. **e**, **f** Immunoblot detection of HA-tagged DOT1L (1-416 aa) retrieved by in vitro-transcribed tRSA-tagged LAMP5-AS1 sections from 293T cell lysates. LAMP5-AS1 (503-1036 nt, 1-1036 nt, and 1-1928 nt) presented significant enrichment. Beta-tubulin or lamina A/C as the negative control. **g** RIP-qPCR detection for the enrichment of FLAG-tagged DOT1L (1-416 aa) on LAMP5-AS1 truncated mutants in 293T cells. LAMP5-AS1 (503-1036 nt) presented significantly higher enrichment. Immunoblotting indicated the successful RIP assay. Error bars reflect ± SEM from three independent experiments. **h** Immunoblot detection of HA-tagged DOT1L (1-416 aa) sections retrieved by in vitro-transcribed tRSA-tagged LAMP5-AS1 (503-1036 nt) from 293T cell lysates. DOT1L (1-407 aa, 360-416 aa, and 1-416 aa) presented significantly higher enrichment of LAMP5-AS1 (503-1036 nt). Lamina A/C as the negative control. **i** RIP-qPCR detection for the enrichment of HA-tagged DOT1L (1-416 aa) sections on LAMP5-AS1 (503-1036 nt) in 293T cells. LAMP5-AS1 (503-1036 nt) was highly enriched in DOT1L (1-407 aa, 360-416 aa, and 1-416 aa)-transfected 293T cell lysates. Error bars reflect ± SEM from three independent experiments. Immunoblotting indicated the successful RIP assay. Lamina A/C as the control

Next, we investigated the mechanism by which LAMP5-AS1 affects the self-renewal capacity of *MLL* leukemia cells. It has been reported that lncRNAs could involve in the regulation of chromatin accessibility [34], transcription [35], splicing [36], translation [37], protein location, and function [38], and the modes of actions are dependent on the sublocation of lncRNAs in cells [22]. Therefore, we first performed subcellular fractionation and fluorescence in situ hybridization (FISH) to detect the nuclear/cytoplasm distribution of LAMP5-AS1 and found that LAMP5-AS1 mainly localized to the nucleus (Additional file 1: Figure S4a and b). We then performed an RNA pull-down assay together with mass spectrometry (MS) to identify proteins interacting with the lncRNA. The antisense sequence of LAMP5-AS1 was utilized as a negative control (Fig. 3a; Additional file 1: Figure S4c). The identified proteins are shown in Additional file 1: Table S6. Among the proteins potentially interacting with LAMP5-AS1, histone methyltransferase DOT1L attracted our interest. DOT1L has been reported to be recruited by MLL fusion proteins, resulting in ectopic H3K79 methylation for epigenetic activation of the *HOXA* genes, *MEIS1* gene, and others [39–42]. DOT1L plays an essential role in the development of *MLL* leukemia. Thus, we speculated that LAMP5-AS1 might function by interacting with DOT1L.

To address this hypothesis, we first confirmed the interaction between LAMP5-AS1 and DOT1L. RNA pull-down followed by western blotting showed that LAMP5-AS1 had a strong interaction with DOT1L (Fig. 3a). The specificity of this RNA-protein interaction was further verified by RNA immunoprecipitation (RIP) of DOT1L-FLAG in *MLL* leukemia cell lines (Fig. 3b; Additional file 1: Figure S4d-f). Moreover, RNA FISH and immunofluorescence (IF) experiments also showed that LAMP5-AS1 colocalized with DOT1L in the cell nucleus (Fig. 3c; Additional file 1: Figure S4g), indicating that LAMP5-AS1 could feasibly interact with DOT1L.

To further explore how LAMP5-AS1 binds to DOT1L and whether it has the potential to influence the histone methyltransferase functions, we next performed experiments with *DOT1L* or *LAMP5-AS1* truncated mutants. We constructed *DOT1L* truncated mutants tagged with FLAG retaining either the methyltransferase active center (1-416 aa) or MLL fusion protein-binding domain or its residual components as previously described [43] (Fig. 3d). Full-length or truncated *DOT1L* were transfected into HEK293T cells with LAMP5-AS1, and potential interactions were detected by RIP. The results showed that the 1-416 aa segment of DOT1L was sufficient to bind LAMP5-AS1 (Fig. 3d). Then, a series of *LAMP5-AS1* truncated mutants were constructed to determine the binding sites of LAMP5-AS1 interacting with DOT1L. These mutant sequences were fused with tRSA, a tRNA scaffold that includes a streptavidin aptamer, for RNA pull-down assay [44, 45]. The results showed that 503-1036 nt of the lncRNA specifically bound to DOT1L (1-416 aa) (Fig. 3e, f), which was further proven by the RIP assay (Fig. 3g). Previous studies have reported that the 390-407 aa region, a Lys-rich domain that is important for DOT1L (1-416aa) catalyzing H3K79 methylation [43]. The RIP and tRSA pull-down assays showed that DOT1L truncated mutants who retained the 390-407 aa region displayed a strong interaction with the 503-1036 nt section of LAMP5-AS1 (Fig. 3h, i). Notably, when we deleted the 390-407 aa segment, there was a drastic decrease in the interaction of DOT1L with LAMP5-AS1, highlighting that LAMP5-AS1 specifically bound to the Lys-rich region of DOT1L catalytic domain (Fig. 3h, i). We wondered whether LAMP5-AS1 affected self-renewal in *MLL* leukemia cells by regulating the methyltransferase activity of DOT1L.

### LAMP5-AS1 affects the methyltransferase activity of DOT1L

**Fig. 4:**
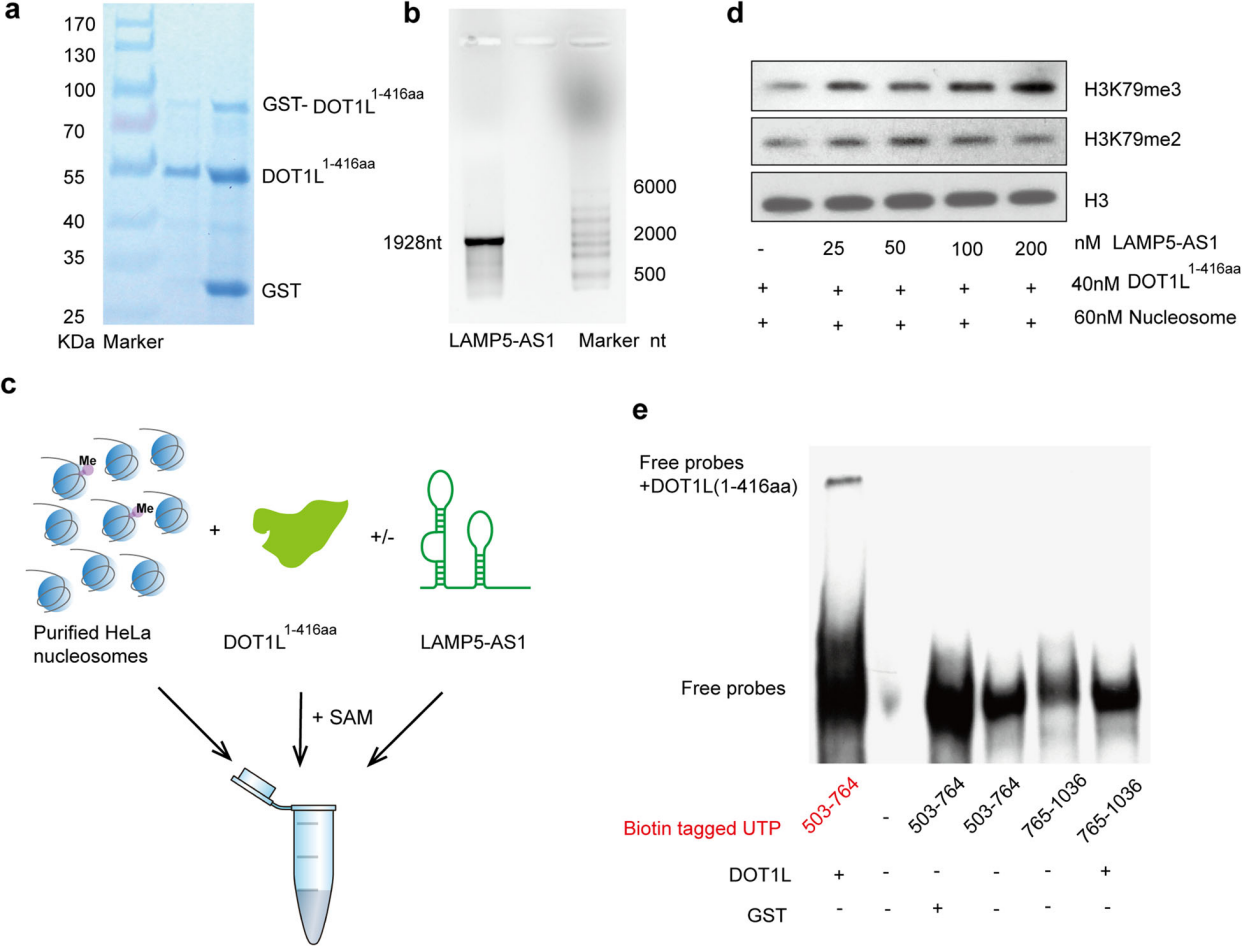
Effect of LAMP5-AS1 on the HMTase activity of DOT1L. **a** GST-tagged DOT1L (1-416 aa) overexpressed in one-shot BL21(DE3)-competent *E*. *coli* cells and purified using GST affinity purification and then digested with thrombin is shown by Coomassie staining. GST-tagged DOT1L (1-416 aa), DOT1L (1-416 aa), and GST are presented. **b** Electrophoresis of RNA on gel containing formaldehyde, showing LAMP5-AS1 results from RNA transcription in vitro. **c** Schematic depiction of the experimental design. **d** Representative HMT assay checked by H3K79me2 and H3K79me3 immunoblotting showed that LAMP5-AS1 enhanced the HMTase activity of DOT1L. Purified HeLa nucleosome was used in the assay. **e** Binding of the active domain (503-764 nt) of LAMP5-AS1 to DOT1L (1-416 aa) measured by electrophoretic mobility shift assay (EMSA)

Cell-free histone methyltransferase (HMTase) assays were performed to determine if LAMP5-AS1 affects DOT1L methyltransferase activity by binding to its Lys-rich region of catalytic domain. Purified LAMP5-AS1 and recombinant GST-tagged 1-416 aa of human DOT1L were produced by in vitro transcription and a prokaryotic expression system, respectively (Fig. 4a, b). The purified 1-416 aa region of human DOT1L alone or in a complex with the full-length LAMP5-AS1, the nucleosomes isolated from HeLa cells, and S-adenosyl-methionine were used in the HMTase assays (Fig. 4c). We found that histones methylated by the DOT1L-LAMP5-AS1 complex had a significantly higher H3K79me3 than those methylated by DOT1L alone, suggesting that the DOT1L-LAMP5-AS1 complex had greater methyltransferase activity and that the lncRNA had the ability to stimulate higher order H3K79 methylation (Fig. 4d). Moreover, slight changes in H3K79me2 were observed between treatments with DOT1L-LAMP5-AS1 or DOT1L alone, which we thought was due to the abundant H3K79me2 background of nucleosomes extracted from HeLa cells (Fig. 4d). We further preformed an electrophoretic mobility shift assay (EMSA) and identified that a direct interaction between LAMP5-AS1 (503-764 nt) and DOT1L (1-416 aa) (Fig. 4e; Additional file 1: Figure S4h). These results demonstrated that LAMP5-AS1 promoted the self-renewal capacity of *MLL* leukemia cells by enhancing the methyltransferase activity of DOT1L.

### LAMP5-AS1 regulates the global patterns of H3K79me2/3 of MLL fusion protein target genes

**Fig. 5:**
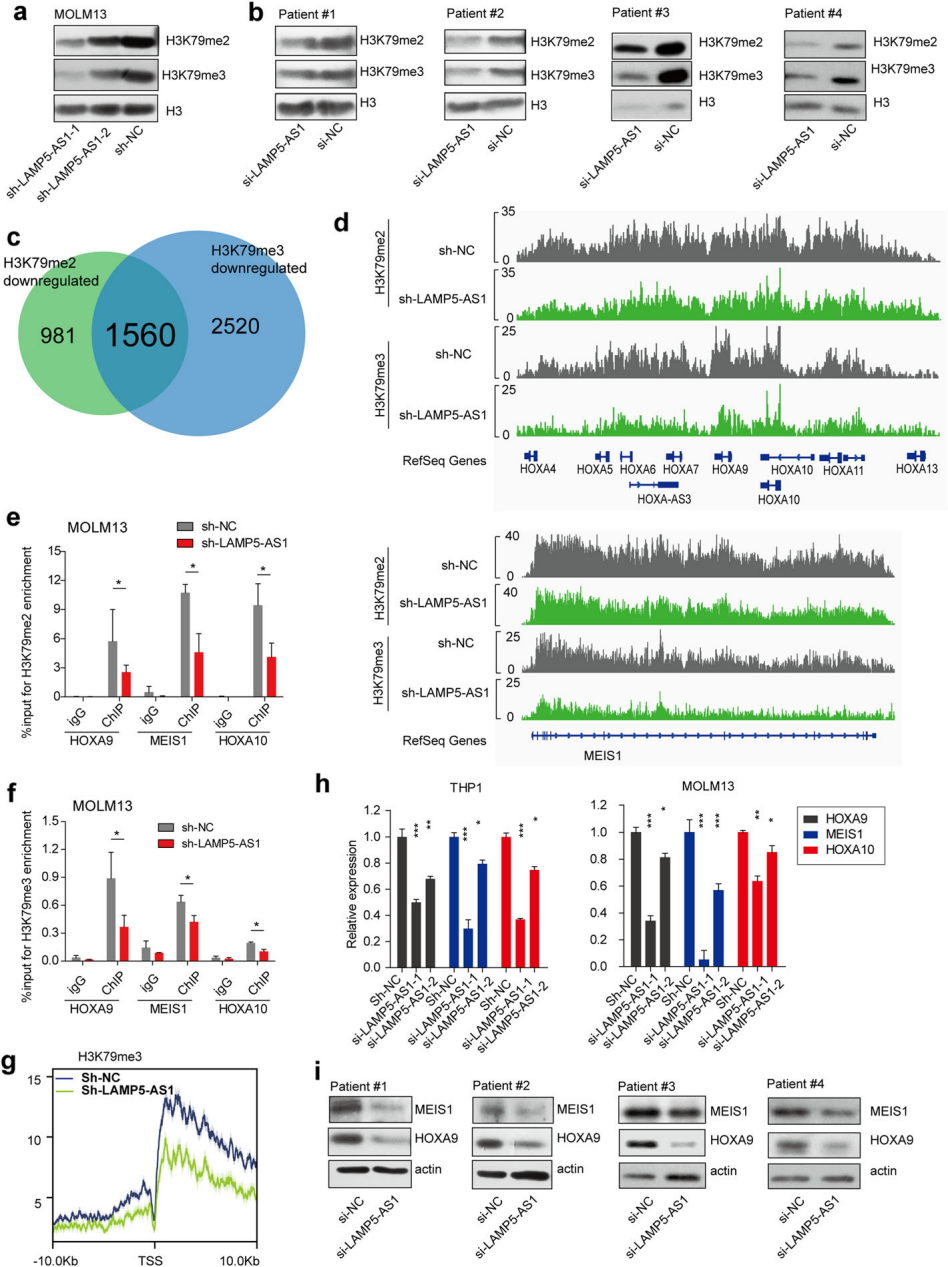
Genomic and epigenomic changes upon LAMP5-AS1 deletion. **a**, **b** Immunoblot analysis of H3K79me2 and H3K79me3 in MOLM13 cells (*MLL-AF9*) (**a**) and 4 primary cells from patients with *MLL* leukemia (**b**) after transduction with LAMP5-AS1 siRNAs or control. **c** Venn diagram shows the overlaying gene sets displaying the loss of H3K79me2 and H3K79me3 marks upon comparison of sh-LAMP5-AS1 with sh-NC data. **d** ChIP-seq profiles of H3K79me2 and H3K79me3 at the *HOXA* gene cluster and *MEIS1* genomic loci in LAMP5-AS1 knockdown (green) compared with control (gray) MOLM13 cells. The *y*-axis scales represent read density per million sequenced reads. **e**, **f** H3K79me2 (**e**) and H3K79me3 (**f**) ChIP-qPCR for the core target genes of the MLL fusion protein in the LAMP5-AS1 knockdown (red) compared with control (gray) established MOLM13 cells. Error bars reflect ± SEM (*p < 0.05) from three independent experiments. **g** Representative meta-analysis plot showing H3K79me3 profile across the + 10 kb to − 10 kb genomic region around the transcription start site (TSS) of MLL-AF9 target genes. Profiles of LAMP5-AS1 knockdown (green) compared with control (blue) MOLM13 cells are presented. **h** The MLL fusion protein target genes *HOXA9*, *HOXA10*, and *MEIS1* with downregulated expression levels upon knockdown of LAMP5-AS1 in *MLL* leukemia cell lines. Error bars reflect ± SEM (*p < 0.05, **p < 0.01; ***p < 0.001) in three independent experiments. **i** Immunoblot analysis of the MLL fusion protein target genes *HOXA9* and *MEIS1* after downregulation of LAMP5-AS1 in 4 primary cells from patients with *MLL* leukemia

Given that LAMP5-AS1 facilitates the methyltransferase activity of DOT1L by directly binding to the N terminal catalytic domain, we asked whether LAMP5-AS1 could affect the genome-wide levels of H3K79me2/3. Notably, LAMP5-AS1 knockdown produced dramatic decrease in H3K79me2 and H3K79me3 levels both in the MOLM13 cells as well as in the primary *MLL* leukemia cells, as assessed by western blotting (Fig. 5a, b), suggesting that LAMP5-AS1 could be a critical factor for higher H3K79-methylated states. We further analyzed H3K79me2 and H3K79me3 profiles by chromatin immunoprecipitation and found that 1560 genes display loss of both H3K79me2 and H3K79me3 marks compared to sh-NC data (Fig. 5c), indicating that LAMP5-AS1 participates in the regulation of genomic or sectional H3K79me2/3 modifications in *MLL* leukemia. Specifically, at the locus of *HOXA* gene cluster, *CDK6* and *MEIS1*, which are the core target genes of MLL fusion proteins, we found significant decreases in H3K79 methylation including a prominent reduction in H3K79me3 and a considerable decrease in H3K79me2 (Fig. 5d; Additional file 1: Figure S5a). The ChIP-qPCR assays also confirmed the downregulation of H3K79me2/3 at *HOXA9*, *HOXA10*, and *MEIS1* gene bodies upon LAMP5-AS1 knockdown (Fig. 5e, f; Additional file 1: Figure S5b). We then conducted a meta-analysis of all 129 MLL-AF9 target genes to assess changes of H3K79me2 and H3K79me3 across the gene body when LAMP5-AS1 was knocked down [14]. Notably, we observed a significant decrease in H3K79me3 profile plot and a considerable reduction of H3K79me2 in LAMP5-AS1 knockdown samples compared to the negative controls (Fig. 5g; Additional file 1: Figure S5c). The results showed that LAMP5-AS1 knockdown could affect the methyltransferase activity of DOT1L and prevent the H3K79me2/3 at the most of MLL fusion target genes in leukemia.

To further demonstrate the effect of LAMP5-AS1 on DOT1L activity, we investigated the expression patterns of the core target genes of MLL fusion protein whose H3K79me2/3 methylation was reduced at gene locus when LAMP5-AS1 was suppressed. The results showed that the expression levels of MLL fusion protein target genes in leukemia, including the key stemness genes in *HOXA* cluster and *MEIS1* [46], were significantly decreased in both primary *MLL* leukemia cells and *MLL* cell lines upon LAMP5-AS1 knockdown compared to cells with control treatment (Fig. 5h, i; Additional file 1: Figure S6a-c). Overexpressing LAMP5-AS1 showed an opposite result (Additional file 1: Figure S6d-f). These observations supported the conclusion that LAMP5-AS1 is essential to promote self-renewal and block differentiation by upregulating H3K79me2/me3 and the transcription of DOT1L ectopic target genes in *MLL* leukemia cells.

### LAMP5-AS1 serves as a predictor of poor outcome in *MLL* leukemia

**Fig. 6:**
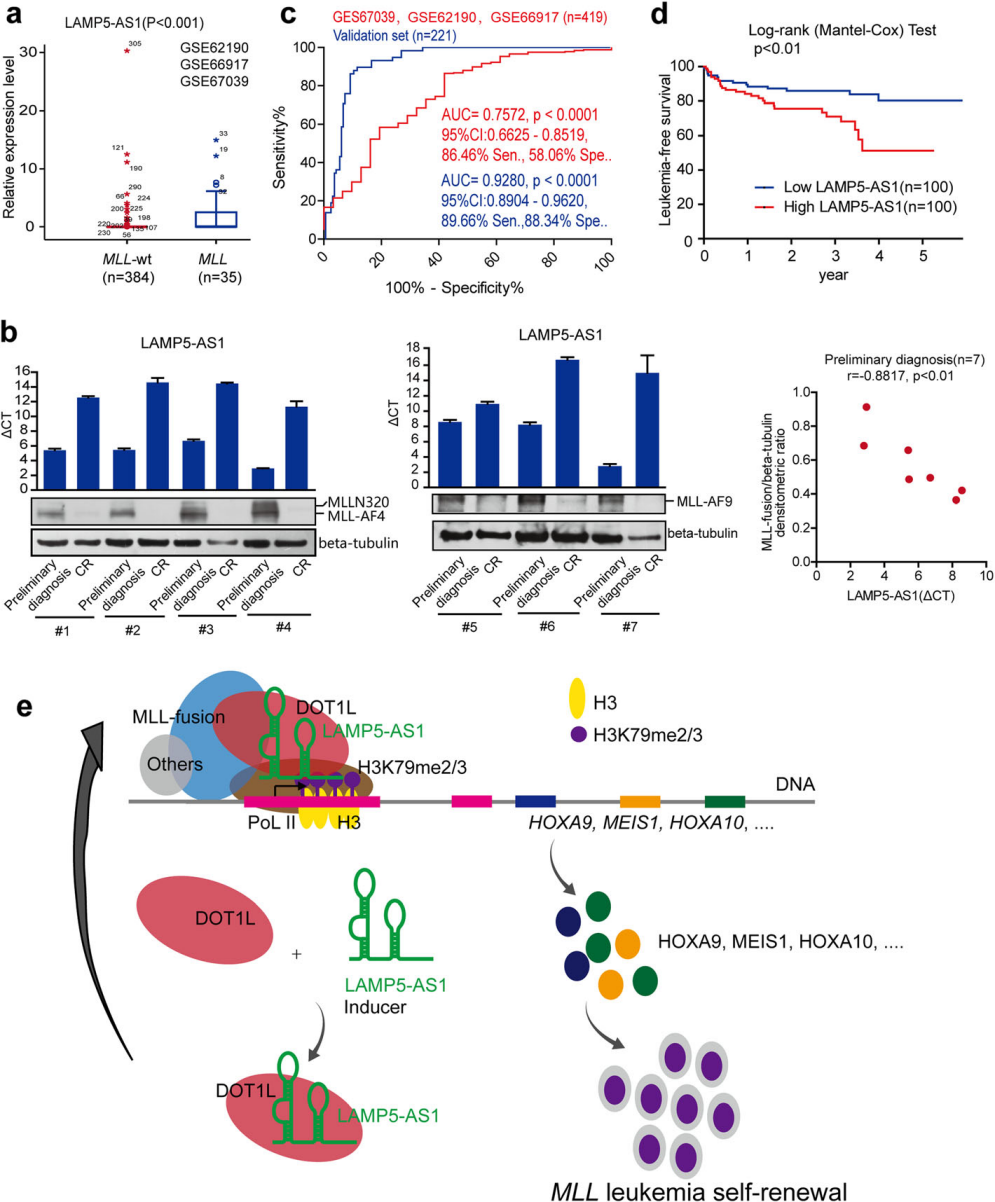
LAMP5-AS1 could serve as a prognostic predictor of *MLL* leukemia. **a** Reanalysis of the GSE62190, GSE66917, and GSE67039 data sets with 419 patient samples classified into *MLL* leukemia and *MLL-wt* subtypes. LAMP5-AS1 expression presented the highest levels in *MLL* leukemia (Mann-Whitney test, *p* < 0.001). **b** LAMP5-AS1 and MLL fusion protein levels in 7 paired *MLL* leukemia patients (initial diagnosis versus complete response, CR), and the MLL fusion protein levels were positively correlated with those of LAMP5-AS1 (△CT) at preliminary diagnosis (Pearson *r* = − 0.8817, *p* < 0.01). Relative expression (ΔCT) was used to quantify LAMP5-AS1 expression relative to a housekeeping gene (GAPDH). **c** ROC curve analysis showed that LAMP5-AS1 had high AUC values of 0.7572 (95% confidence interval (CI) 0.6625–0.8519) and 0.9280 (95% CI 0.8904–0.9620, *p* < 0.001) in the GSE62190, GSE66917, and GSE67039 data sets (*n* = 35 for *MLL* leukemia and *n* = 384 for *MLL-*wt) and validation set (*n* = 58 for *MLL* and *n* = 163 for *MLL-*wt), respectively, with considerably significant sensitivity (sen.) and specificity (spe.) at the optimal cutoff point calculated by Youden’s index. **d** The 5-year leukemia-free survival of patients with a high expression level of LAMP5-AS1 is less than that of patients with a low LAMP5-AS1 level in *MLL* leukemia (*n* = 200, *p* < 0.01). **e** A working model proposed for the specific activation of DOT1L/H3K79 methyltransferase by LAMP5-AS1 binding to regulate *MLL* leukemia self-renewal

Finally, we evaluated the clinical relevance of the lncRNA. We reanalyzed the microarray studies of 419 patient samples (GSE62190, GSE66917, and GSE67039) classified into *MLL* leukemia and *MLL-*wt subtypes [47]. The results clearly showed that LAMP5-AS1 was differentially expressed among these groups, with the highest expression levels in *MLL* leukemia (Fig. 6a), which is similar to the outcome detected in our data set shown in Fig. 1a. We further tested 7 paired *MLL* leukemia patients, containing 4 *MLL-AF4* and 3 *MLL-AF9*, at preliminary diagnosis and CR. Similarly, the expression of LAMP5-AS1, which has a prominent positive correlation with MLL fusion protein levels, was significantly decreased in CR samples compared to preliminary diagnosis samples (Fig. 6b). These results may suggest that special upregulation LAMP5-AS1 could be a useful biomarker for *MLL* leukemia. Subsequently, we evaluated the clinical value of the aberrantly expressed LAMP5-AS1. A receiver operating characteristic (ROC) curve analysis was performed to distinguish patients with *MLL* leukemia from those without, and the results showed that LAMP5-AS1 achieved a high AUC value in both the GSE62190, GSE66917, and GSE67039 data set (*n* = 35 for *MLL* and *n* = 384 for *MLL-*wt) and our sample validation set (*n* = 58 for *MLL* and *n* = 163 for *MLL-*wt), with a considerably significant sensitivity and specificity at the cutoff point (Fig. 6c). A leukemia-free survival analysis demonstrated that higher levels of LAMP5-AS1 may lead to reduced 5-year leukemia-free survival (Fig. 6d). From the above, we showed that high expression of LAMP5-AS1 was significantly associated with poor outcomes of *MLL* leukemia patients, suggesting that this lncRNA could serve as a biomarker for the diagnosis and prognosis of *MLL* leukemia in the future.

## Discussion

*MLL* leukemia is one of the most aggressive acute leukemia subtypes and has an apex self-renewal capacity [6, 8]. It remains a challenge to directly inhibit rearranged *MLL* itself in view of its multiple fusion partners [1, 48, 49]. Nevertheless, the dependency of the *MLL* fusion-driven gene expression program on the DOT1L pathway provides potential therapeutic opportunities for *MLL* leukemia [46, 48, 50, 51]. Therefore, further study of the DOT1L biology in *MLL* leukemias could offer alternative strategies to inhibit the acquired self-renewal in *MLL* leukemia stem and progenitor cells by targeting DOT1L pathways. In this study, we found that the lncRNA LAMP5-AS1, which is specifically expressed in *MLL* leukemias, serves as an effector molecule to upregulate DOT1L methyltransferase activity by binding to its active center. We demonstrated that LAMP5-AS1 knockdown triggered a decreased H3K79 methylation state on the locus of the *HOXA* genes and *MEIS1*, which remarkably inhibited *MLL* leukemia cell self-renewal and promoted differentiation (Fig. 6e)*.* We are the first to demonstrate that lncRNAs can play a crucial role in the progression of *MLL* leukemias and can directly influence the enzyme activity of H3 methyltransferase as effector molecules like coenzymes. We showed that LAMP5-AS1 is crucial for self-renewal in *MLL* leukemia cells and may be a valuable therapeutic target for *MLL* leukemia treatment.

LncRNAs have been illustrated to play a pivotal role in the progression of cell fate and cancer development, including hematopoiesis and leukemogenesis [17, 21, 24, 52]. They exert their functions via cotranscriptional regulation, gene expression modulation, scaffolding of protein complexes, and pairing with other RNAs [22, 53]. Notably, recent studies showed that lncRNAs modulated gene expression as epigenetic histone modifiers [54, 55]. For instance, lncRNA HOTTIP interacts with WDR5 to recruit the MLL H3K4 methylase complex to facilitate H3K4me3 [56]. LncRNA XIST recruits polycomb repressive complex 2 to induce H3K27me3 and silence the X chromosome [57]. Nevertheless, in most studies on lncRNA functions in the regulation of epigenetic histone modification, lncRNAs are described as simple scaffolds to regulate the localization of activating or repressive chromatin modification machinery [54, 55]. However, little is known about whether lncRNAs could directly regulate the activity of modification machines that would influence modification of the whole chromatin. In this study, we revealed that LAMP5-AS1 enhanced the methyltransferase activity of DOT1L by directly binding to its activity site. LAMP5-AS1 knockdown significantly reduced the global H3K79me2/me3 levels in *MLL* leukemia cells, resulting in significantly decreased expression of the stem gene *HOXA* cluster and *MEIS1* and decreased cell self-renewal capacity*.* We demonstrate a novel function of lncRNAs to modulate global epigenetic histone modification by directly regulating enzymatic activity as effector molecules like coenzymes, rather than as simple scaffolds. The results highlight the extensive functions of lncRNAs on the regulation of epigenetic histone modification.

Recently, the positive feedback mechanisms in which posttranscriptional regulation is connected to transcriptional programs highlight a novel way to sustain the program of leukemic stem and progenitor cells [58]. Interestingly, we showed that LAMP5-AS1 interacted with the methyltransferase active center of the mature DOT1L protein and could enhance its enzyme activity at the posttranslational level. LAMP5-AS1 knockdown significantly decreased global H3K79me2/me3 levels, which sharply inhibited the expression of the stem genes in the *HOXA* cluster and *MEIS1* at the transcriptional level. These results suggest that the LAMP5-AS1-DOT1L axis can partially integrate the posttranslational regulation into the transcription program to sustain self-renewal capacity in *MLL* leukemia.

DOT1L, a H3K79 methyltransferase without the SET domain that is found in most histone lysine methyltransferases, requires a more complicated complex to perform selective H3K79 methylation at different levels [15]. Importantly, LAMP5-AS1 has been reported to be specifically highly expressed in *MLL* leukemia cells, while it has an extra low expression level in *MLL*-wt leukemia and general blood cells [26]. Thus, we speculated that the MLL fusion protein purposely enhanced the methyltransferase activity of DOT1L through upregulation of LAMP5-AS1 to obtain a high self-renewal capacity. A recent study also showed that a cofactor of DOT1L AF10, which is highly expressed in mouse LSK cells, can facilitate the methyltransferase activity of DOT1L to obtain higher order H3K79 methylation [14]. Recently, AF10 has been verified to bind to CC0 (amino acids 483 to 502) and a longer CC1 helix (amino acids 510 to 549) of DOT1L in *MLL-AF10* leukemia [59]; both domains are near to the K-rich region that LAMP5-AS1 interacted with, suggesting that LAMP5-AS1 and AF10 might cooperate to enhance the methyltransferase activity of DOT1L in *MLL* leukemia. Taken together, these results explain the specific oncogenicity of the ubiquitously expressed DOT1L from another perspective. Although there is a new controversy about whether DOT1L is necessary for *MLL* LSC maintenance [60], the essential role of DOT1L in sustaining *MLL* leukemia cell self-renewal ability is undisputed [14, 61]. Due to the complicated pathogenesis of *MLL* leukemia and the potential limitation on direct DOT1L-targeted strategies, optional approaches with more precise targeted therapies, including lncRNA and other drugs highly specific to LSC, are therefore required, particularly as combination regimens to treat different clinical responses.

## Conclusions

In summary, our results demonstrated that lncRNA LAMP5-AS1 played an important role specifically in the self-renewal program and differentiation block in *MLL* leukemia cells. Our studies indicated the importance of LAMP5-AS1 to sustain the high enzymatic activity of DOT1L in *MLL* leukemia and presented the possibility that LAMP5-AS1 could be a valuable therapeutic target for *MLL* leukemia treatment.

## Supplementary information

**Additional file 1. Figure S1.** Identification of LAMP5-AS1 transcripts. **a** Agarose gel for the 5’ and 3’ RACE identified LAMP5-AS1 transcripts in THP1 cells. Two 5’-ends and one 3’-end of LAMP5-AS1 variant cDNA in cells were identified by the nested PCR. Schematic depiction of the two LAMP5-AS1 transcripts (bottom). **b** qRT-PCR for the relative expression of the two different LAMP5-AS1 transcripts. **Figure S2.** Impact of LAMP5-AS1 on primary *MLL* leukemia differentiation. **a** LAMP5-AS1 was highly expressed in all *MLL* leukemia cell lines labeled by * (analyzed by △CT). **b** qRT-PCR analysis for LAMP5-AS1 knockdown in 4 primary cells from patients with *MLL* leukemia, including three ALL with MLL-AF9 and MLL-AF4 and an AML with MLL-AF10, respectively, after transduction with LAMP5-AS1 siRNAs or control. Error bars reflect ± SEM (*, *p* < 0.05, **, *p* < 0.01) in three independent experiments. **c** Representative graph for the flow cytometric analysis of the CD19+, CD11b+, or CD14+ cell populations in the primary *MLL* leukemia cells. **d** Representative graph for the flow cytometric analysis of the CD34+ cell populations in primary *MLL* leukemia cells. Histogram plots show the statistical values. Error bars reflect ± SEM (*, *p* < 0.05, **, *p* < 0.01) in three independent experiments. **Figure S3.** LAMP5-AS1 plays a role in *MLL* leukemia cell maintenance. **a, b** qRT-PCR analysis for LAMP5-AS1 knockdown in *MLL* leukemia cells, after transduction with LAMP5-AS1 siRNAs or control (**a**) and LAMP5-AS1 shRNAs or control (**b**). Error bars reflect ± SEM (**, *p* < 0.01; ***, *p* < 0.001) in three independent experiments. **c-e** Representative flow cytometry graphs showing the CD14 (**c**), CD11b (**d**), and CD19 (**e**) cell populations in *MLL* leukemia cells treated with LAMP5-AS1 knockdown relative to those levels in control. The values were analyzed by Error bars reflect ± SEM (*,*p* < 0.05, **,*p* < 0.01,***, *p* < 0.001) in three independent experiments. **f** Morphology of colonies of MLL leukemia cells 10 days upon shRNA-mediated knockdown of LAMP5-AS1. Scale bars, 100 μm. Error bars reflect ± SEM (***, *p* < 0.001) in three independent experiments. **Figure S4.** Identification of LAMP5-AS1 binding to DOT1L in cell nucleus. **a** We fractionated the nucleus and cytoplasm from the THP1 cells and found that LAMP5-AS1 predominantly localizes to the cell nucleus, with NEAT1 as a nuclear marker and hY1 as a cytoplasmic marker. Error bars reflect ± SEM (***, *p* < 0.001) in three independent experiments. **b** RNA FISH showing most of LAMP5-AS1 localizes in the nuclei of *MLL* leukemia cells. Scale bars, 5 μm. **c** Agarose gel showing the templates of LAMP5-AS1 and LAMP5-AS1 antisense in the RNA-pull-down assay. **d** Agarose gel showing the PCR template of DOT1L. **e** Western blotting of DOT1L-N-FLAG in the products of RIP, with beta-tubulin as the negative control. Cell lysis harvested from the DOT1L-N-FLAG stably expressed THP1 cells. **f** RIP of DOT1L-FLAG in MOLM13 indicating that LAMP5-AS1 was significantly enriched compared with U6, actin, and GAPDH. **g** RNA FISH and IF experiments showed that LAMP5-AS1 co-localizes with DOT1L in the nuclei of MV4-11 cells. Scale bars, 5 μm. **h** Agarose formaldehyde gel showing the *in vitro* RNA transcription of LAMP5-AS1 sections. Biotin labeled UTP was added in the reaction. **Figure S5.** Epigenomic changes upon LAMP5-AS1 knockdown. **a** ChIP-seq profiles of H3K79me2 and H3K79me3 at the *CDK6* genomic loci in LAMP5-AS1-knockdown (green) compared with control (gray) MOLM13 cells. The y-axis scales represent read density per million sequenced reads. **b** H3K79me2(left) and H3K79me3(right) ChIP-qPCR for the core target genes of MLL fusion protein in the LAMP5-AS1 knockdown (red) compared with control (gray) established MOLM13 cells. Error bars reflect ± SEM (*, *p* < 0.05) from three independent experiments. **c** Representative meta-analysis plot showing H3K79me2 profile across the +10 kb to -10 kb genomic region around the TSS of MLL-AF9 target genes. Profiles of LAMP5-AS1-knockdown (green) compared with control (blue) MOLM13 cells are presented. **Figure S6.** Genomic changes upon LAMP5-AS1 knockdown or overexpression. **a** qRT-PCR analysis determined that the expression levels of the MLL fusion protein target genes including *HOXA9, HOXA10* and *MEIS1* were decreased upon LAMP5-AS1 knockdown in MV4-11 cells. Error bars reflect ± SEM (*, *p* < 0.05, **, *p* < 0.01; ***, *p* < 0.001) in three independent experiments. **b** qRT-PCR analysis determined that the expression levels of the MLL fusion protein target genes including *HOXA9, HOXA10* and *MEIS1* were decreased upon LAMP5-AS1 knockdown in 4 primary *MLL* leukemia cells. Error bars reflect ± SEM (*, *p* < 0.05, **, *p* < 0.01; ***, *p* < 0.001) in three independent experiments. **c** Western blotting for the protein levels of HOXA9 and Mesi1 in *MLL* leukemia cells transduced by LAMP5-AS1 siRNA and control. **d** Overexpression of LAMP5-AS1 transcript 1 in *MLL* leukemia cells (MOLM13, MV4-11, and THP1). **e** qRT-PCR analysis determined that the expression levels of the MLL fusion protein target genes including *HOXA9, HOXA10* and *MEIS1* were increased in *MLL* leukemia cell lines treated with LAMP5-AS1 overexpression. Error bars reflect ± SEM (*, *p* < 0.05, **, *p* < 0.01; ***, *p* < 0.001) in three independent experiments. **f** Immunoblot showing the protein levels of HOXA9 and Mesi1 upregulated upon overexpression of LAMP5-AS1 in *MLL* leukemia cell lines. **Table S1.** Patient demographics and clinicopathologic features. **Table S2.** Demographics and clinicopathologic features of primary *MLL* leukemia patient samples. **Table S3.** The primers used in this work. **Table S4.** siRNA/shRNA. **Table S5.** ALL of the antibodies and regents used in this study. Table S6. MS of proteins from LAMP5-AS1 pull down.

## Data Availability

The material supporting the conclusion of this study has been included within the article.

## Abbreviations

lncRNA: Long noncoding RNA
*LAMP5*: Lysosome-associated membrane protein 5
LAMP5-AS1: *LAMP5* antisense 1
*MLL*: Mixed-lineage leukemia
H3K79: Histone 3 lysine 79
LSC: Leukemia stem cells
*MLL-wt*: Wild-type MLL
FISH: Fluorescence in situ hybridization
CCK8: Cell Counting Kit
siRNAs: Small interfering RNAs
shRNA: Short hairpin RNA
H&E: Hematoxylin and eosin
qRT-PCR: Quantitative reverse transcription-PCR
*HOXA*: Homeobox A
RACE: Rapid amplification of cDNA ends
ROC: Receiver operating characteristic
IMDM: Iscove’s modified Dulbecco medium
FBS: Fetal bovine serum
ELDA: Limiting dilution analysis
MS: Mass spectrometry
RIP: RNA immunoprecipitation
IF: Immunofluorescence
tRSA: tRNA scaffold that includes a streptavidin aptamer
HMTase: Histone methyltransferase
EMSA: Electrophoretic mobility shift assay
GEO: Gene expression omnibus
ChIP-seq: Chromatin immunoprecipitation sequencing
CR: Complete remission
CC: Coiled-coil domain
RT: Room temperature
