# Correction to: The lncRNA LAMP5-AS1 drives leukemia cell stemness by directly modulating DOT1L methyltransferase activity in MLL leukemia

**DOI:** 10.1186/s13045-020-00967-2

**Published:** 2021-03-11

**Authors:** Wen-Tao Wang, Tian-Qi Chen, Zhan-Cheng Zeng, Qi Pan, Wei Huang, Cai Han, Ke Fang, Lin-Yu Sun, Qian-Qian Yang, Dan Wang, Xue-Qun Luo, Yu-Meng Sun, Yue-Qin Chen

**Affiliations:** 1 MOE Key Laboratory of Gene Function and Regulation, State Key Laboratory for Biocontrol, School of Life Sciences, Sun Yat-Sen University, Guangzhou, 510275 China; 2 Sun Yat-Sen University Cancer Center, State Key Laboratory of Oncology in South China, Guangdong, 510060 China; 3 The First Affiliated Hospital of Sun Yat-Sen University, Guangzhou, 510080 China

## Correction to: Journal of Hematology & Oncology (2020) 13:78 10.1186/s13045-020-00909-y

The original article [1] contains an error in Fig. 6b for the image of western blot panels.

**Fig. 6:**
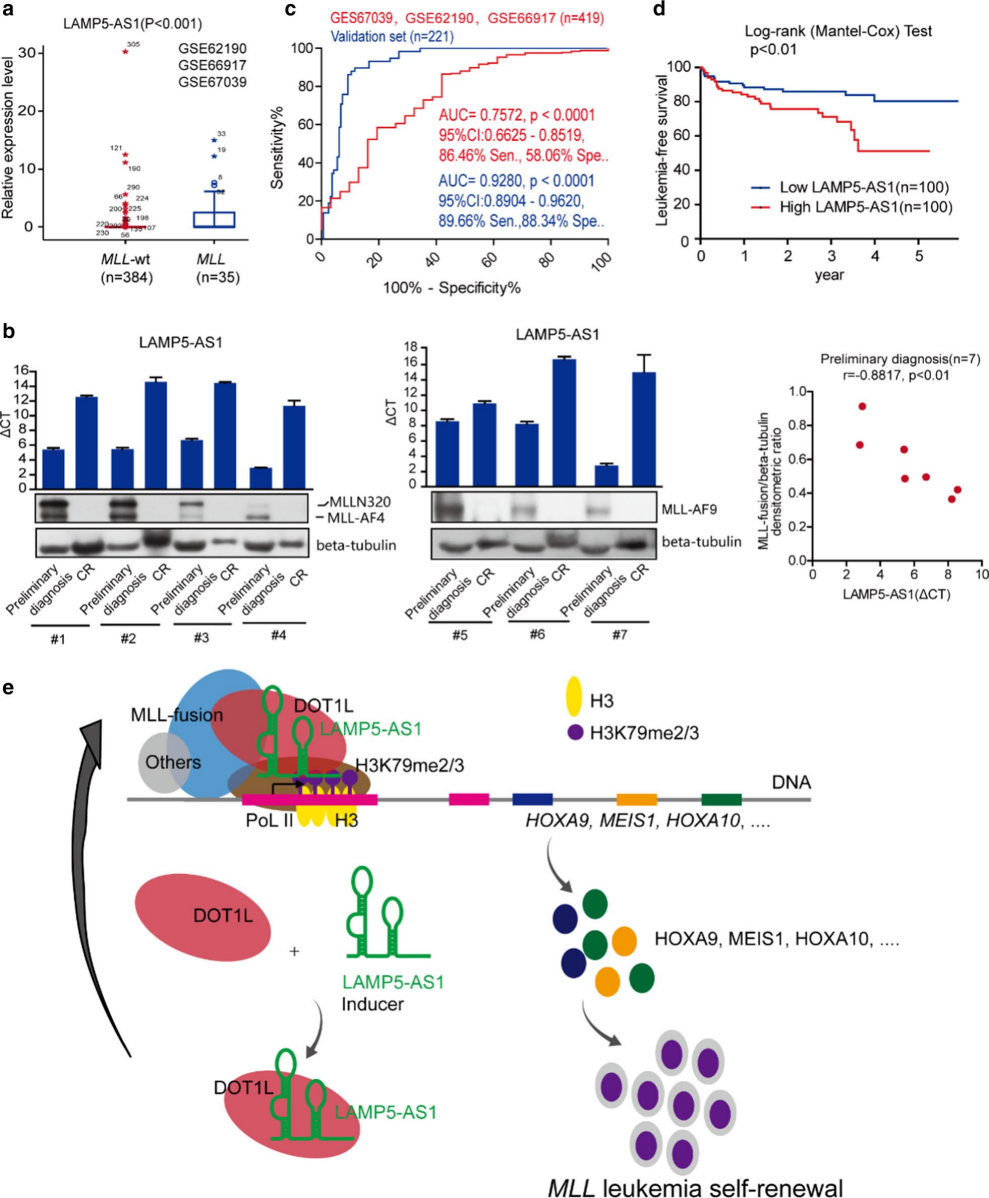
LAMP5-AS1 could serve as a prognostic predictor of *MLL* leukemia. **a** Reanalysis of the GSE62190, GSE66917 and GSE67039 data sets with 419 patient samples classified into *MLL* leukemia and *MLL-wt* subtypes. LAMP5-AS1 expression presented the highest levels in *MLL* leukemia. (Mann–Whitney test,* p* < 0.001). **b** LAMP5-AS1 and MLL fusion protein levels in 7 paired *MLL* leukemia patients (initial diagnosis versus complete response, CR), and the MLL fusion protein levels were positively correlated with those of LAMP5-AS1 (△CT) at preliminary diagnosis (Pearson r = -0.8817, * p* < 0.01). Relative expression (ΔCT) was used to quantify LAMP5-AS1 expression relative to a housekeeping gene (GAPDH). **c** ROC curve analysis showed that LAMP5-AS1 had high AUC values of 0.7572 (95% confidence interval (CI): 0.6625–0.8519) and 0.9280 (95% CI: 0.8904–0.9620, * p* < 0.001) in the GSE62190, GSE66917 and GSE67039 data sets (n = 35 for *MLL* leukemia and n = 384 for *MLL-*wt) and validation set (n = 58 for *MLL* and n = 163 for *MLL-*wt), respectively, with considerably significant sensitivity (sen.) and specificity (spe.) at the optimal cutoff point calculated by Youden’s index. **d** The 5-year leukemia-free survival of patients with a high expression level of LAMP5-AS1 is less than that of patients with a low LAMP5-AS1 level in *MLL* leukemia (n = 200, * p* < 0.01). **e** A working model proposed for the specific activation of DOT1L/H3K79 methyltransferase by LAMP5-AS1 binding to regulate *MLL* leukemia self-renewal

The correct presentation of Fig. 6b is shown below.
