# Supplementary material for: The lncRNA LAMP5-AS1 drives leukemia cell stemness by directly modulating DOT1L methyltransferase activity in MLL leukemia
Source: J Hematol Oncol. 2020 Jun 17;13:78. doi: 10.1186/s13045-020-00909-y (PMC7302350; doi:10.1186/s13045-020-00909-y)
Supplement: Supplementary file 1 — Additional file 1. Figure S1. Identification of LAMP5-AS1 transcripts. a Agarose gel for the 5’ and 3’ RACE identified LAMP5-AS1 transcripts in THP1 cells. Two 5’-ends and one 3’-end of LAMP5-AS1 variant cDNA in cells were identified by the nested PCR. Schematic depiction of the two LAMP5-AS1 transcripts (bottom). b qRT-PCR for the relative expression of the two different LAMP5-AS1 transcripts. Figure S2. Impact of LAMP5-AS1 on primary MLL leukemia differentiation. a LAMP5-AS1 was highly expressed in all MLL leukemia cell lines labeled by * (analyzed by △CT). b qRT-PCR analysis for LAMP5-AS1 knockdown in 4 primary cells from patients with MLL leukemia, including three ALL with MLL-AF9 and MLL-AF4 and an AML with MLL-AF10, respectively, after transduction with LAMP5-AS1 siRNAs or control. Error bars reflect ± SEM (*, p < 0.05, **, p < 0.01) in three independent experiments. c Representative graph for the flow cytometric analysis of the CD19+, CD11b+, or CD14+ cell populations in the primary MLL leukemia cells. d Representative graph for the flow cytometric analysis of the CD34+ cell populations in primary MLL leukemia cells. Histogram plots show the statistical values. Error bars reflect ± SEM (*, p < 0.05, **, p < 0.01) in three independent experiments. Figure S3. LAMP5-AS1 plays a role in MLL leukemia cell maintenance. a, b qRT-PCR analysis for LAMP5-AS1 knockdown in MLL leukemia cells, after transduction with LAMP5-AS1 siRNAs or control (a) and LAMP5-AS1 shRNAs or control (b). Error bars reflect ± SEM (**, p < 0.01; ***, p < 0.001) in three independent experiments. c-e Representative flow cytometry graphs showing the CD14 (c), CD11b (d), and CD19 (e) cell populations in MLL leukemia cells treated with LAMP5-AS1 knockdown relative to those levels in control. The values were analyzed by Error bars reflect ± SEM (*,p < 0.05, **,p < 0.01,***, p < 0.001) in three independent experiments. f Morphology of colonies of MLL leukemia cells 10 days upon shRNA-mediated knockdo [file 13045_2020_909_MOESM1_ESM.docx]

Supplementary Materials


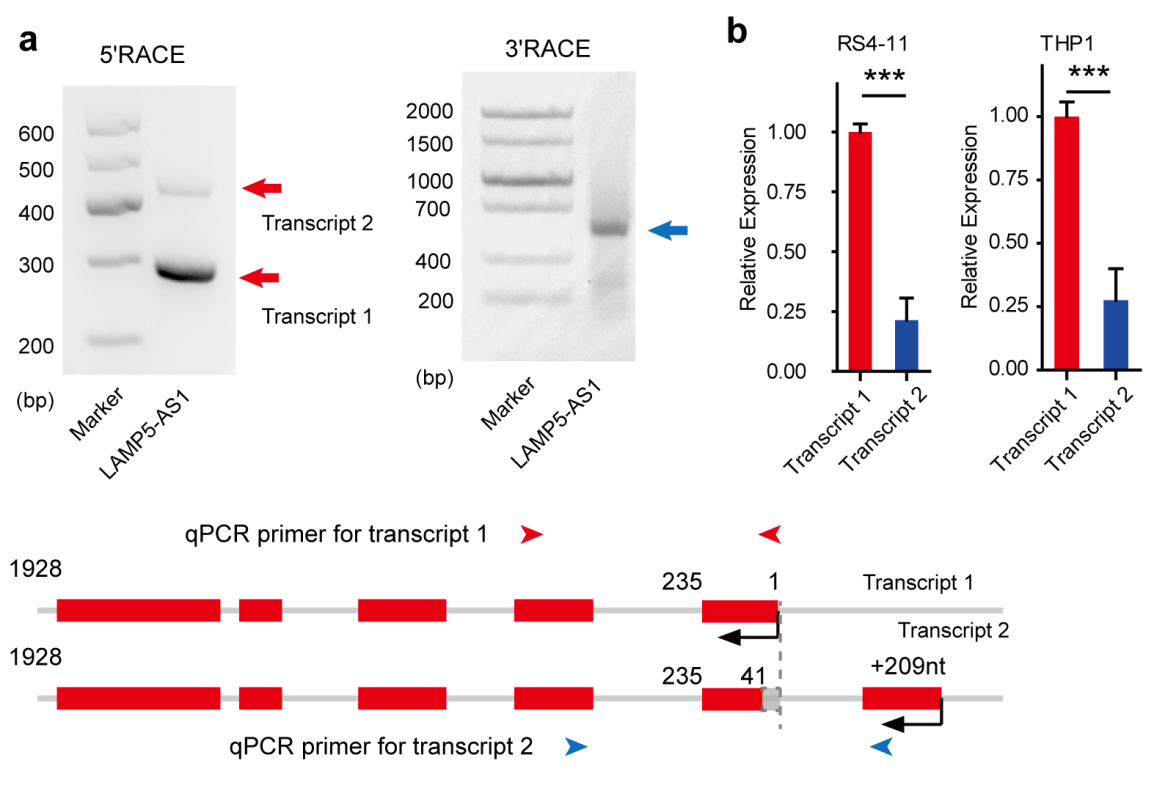


**Figure S1. Identification of** **LAMP5-AS1 transcripts. a** Agarose gel for the 5’ and 3’ RACE identified LAMP5-AS1 transcripts in THP1 cells. Two 5’-ends and one 3’-end of LAMP5-AS1 variant cDNA in cells were identified by the nested PCR. Schematic depiction of the two LAMP5-AS1 transcripts (bottom). **b** qRT-PCR for the relative expression of the two different LAMP5-AS1 transcripts.


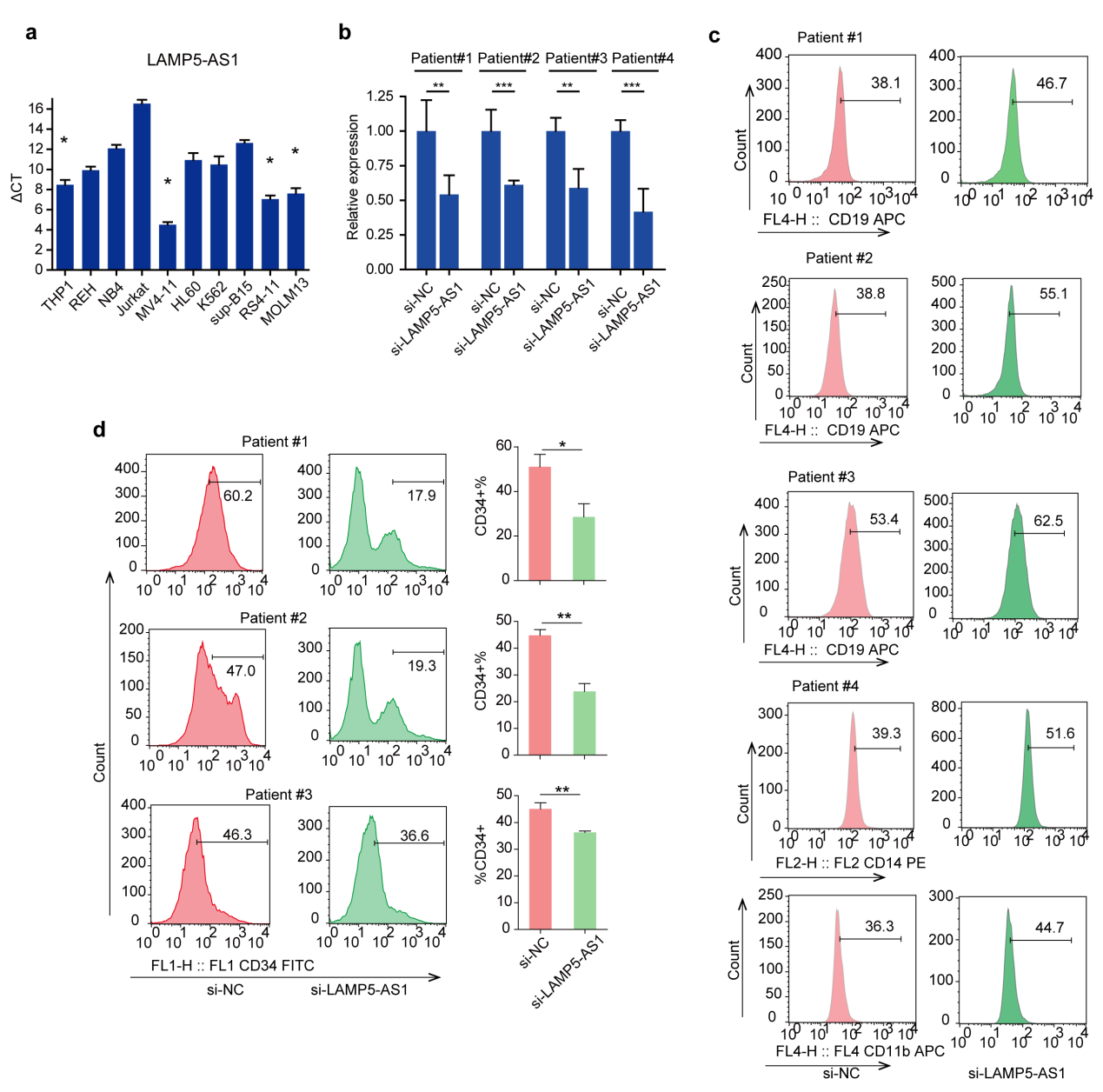


**Figure S2. Impact of LAMP5-AS1 on primary *MLL* leukemia differentiation.**

**a** LAMP5-AS1 was highly expressed in all *MLL* leukemia cell lines labeled by * (analyzed by △CT). **b** qRT-PCR analysis for LAMP5-AS1 knockdown in 4 primary cells from patients with *MLL* leukemia, including three ALL with MLL-AF9 and MLL-AF4 and an AML with MLL-AF10, respectively, after transduction with LAMP5-AS1 siRNAs or control. Error bars reflect ± SEM (*, p<0.05, **, p<0.01) in three independent experiments. **c** Representative graph for the flow cytometric analysis of the CD19+, CD11b+, or CD14+ cell populations in the primary *MLL* leukemia cells. **d** Representative graph for the flow cytometric analysis of the CD34+ cell populations in primary *MLL* leukemia cells. Histogram plots show the statistical values. Error bars reflect ± SEM (*, p<0.05, **, p<0.01) in three independent experiments.


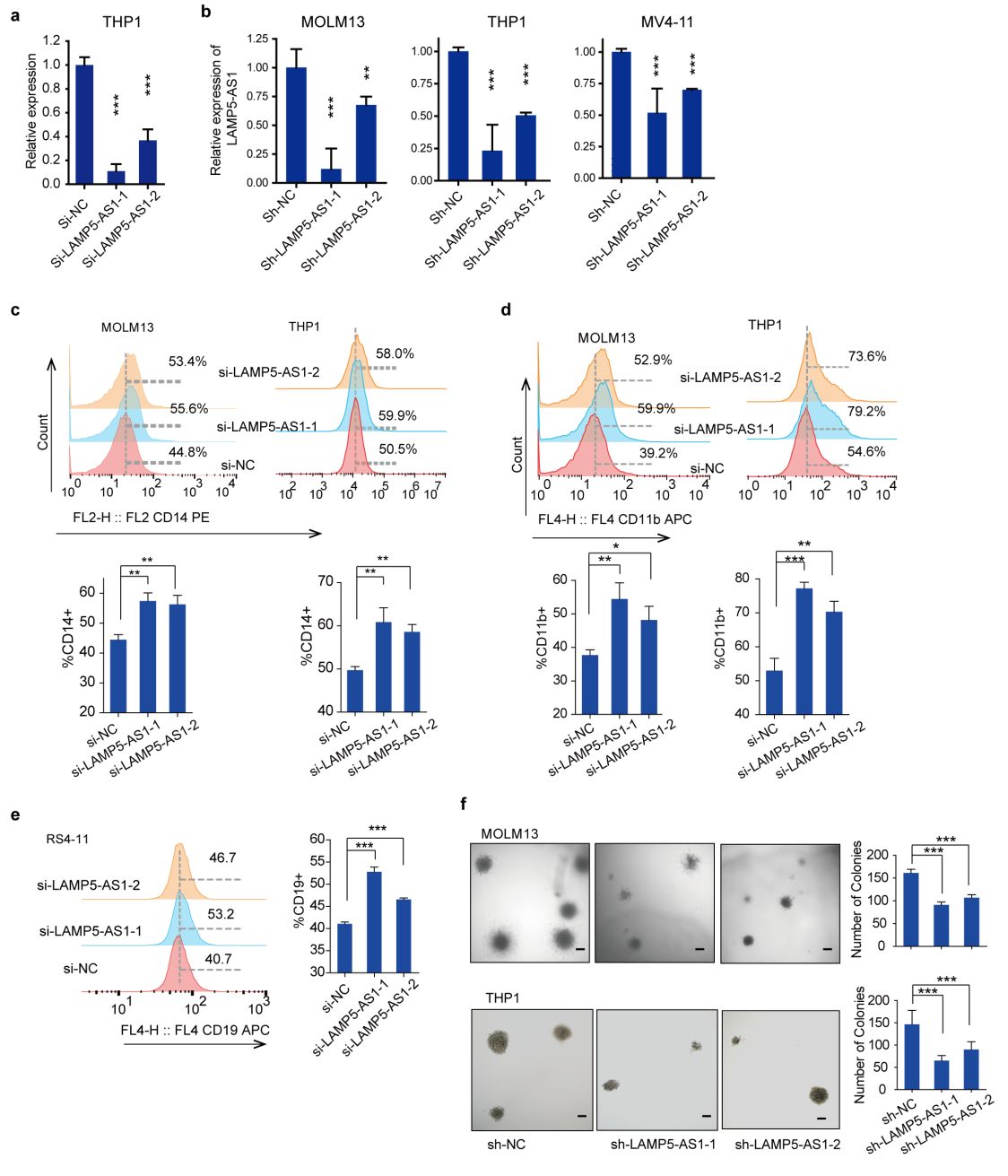


**Figure S3. LAMP5-AS1 plays a role in *MLL* leukemia cell maintenance. a, b** qRT-PCR analysis for LAMP5-AS1 knockdown in *MLL* leukemia cells, after transduction with LAMP5-AS1 siRNAs or control (**a**) and LAMP5-AS1 shRNAs or control (**b**). Error bars reflect ± SEM (**, p<0.01; ***, p<0.001) in three independent experiments. **c-e** Representative flow cytometry graphs showing the CD14 (**c**), CD11b (**d**), and CD19 (**e**) cell populations in *MLL* leukemia cells treated with LAMP5-AS1 knockdown relative to those levels in control. The values were analyzed by Error bars reflect ± SEM (*,p<0.05, **,p<0.01,***, p<0.001) in three independent experiments. **f** Morphology of colonies of MLL leukemia cells 10 days upon shRNA-mediated knockdown of LAMP5-AS1. Scale bars, 100 μm. Error bars reflect ± SEM (***, p<0.001) in three independent experiments.


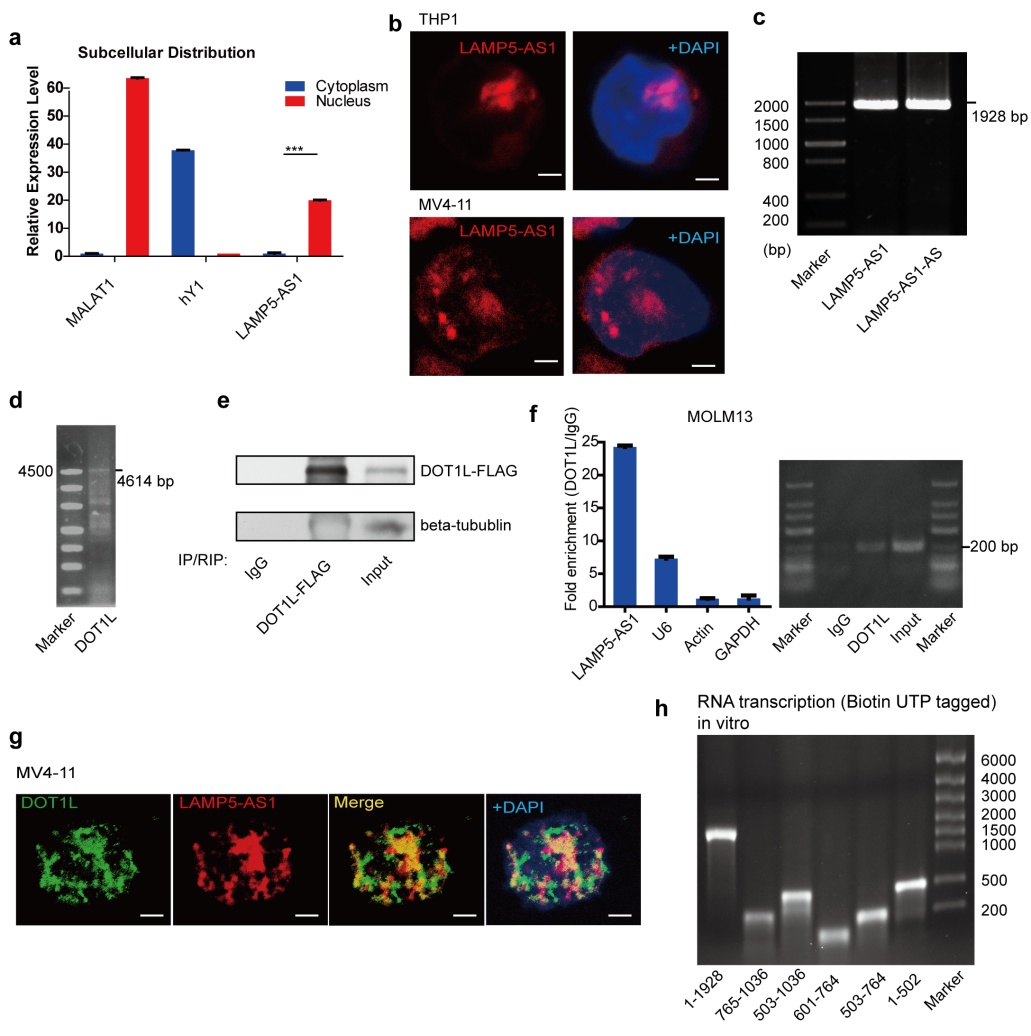


**Figure S4. Identification of LAMP5-AS1 binding to DOT1L in cell nucleus. a** We fractionated the nucleus and cytoplasm from the THP1 cells and found that LAMP5-AS1 predominantly localizes to the cell nucleus, with NEAT1 as a nuclear marker and hY1 as a cytoplasmic marker. Error bars reflect ± SEM (***, p<0.001) in three independent experiments. **b** RNA FISH showing most of LAMP5-AS1 localizes in the nuclei of *MLL* leukemia cells. Scale bars, 5 μm. **c** Agarose gel showing the templates of LAMP5-AS1 and LAMP5-AS1 antisense in the RNA-pull-down assay.

**d** Agarose gel showing the PCR template of DOT1L. **e** Western blotting of DOT1L-N-FLAG in the products of RIP, with beta-tubulin as the negative control. Cell lysis harvested from the DOT1L-N-FLAG stably expressed THP1 cells. **f** RIP of DOT1L-FLAG in MOLM13 indicating that LAMP5-AS1 was significantly enriched compared with U6, actin, and GAPDH. **g** RNA FISH and IF experiments showed that LAMP5-AS1 co-localizes with DOT1L in the nuclei of MV4-11 cells. Scale bars, 5 μm. **h** Agarose formaldehyde gel showing the *in vitro* RNA transcription of LAMP5-AS1 sections. Biotin labeled UTP was added in the reaction.


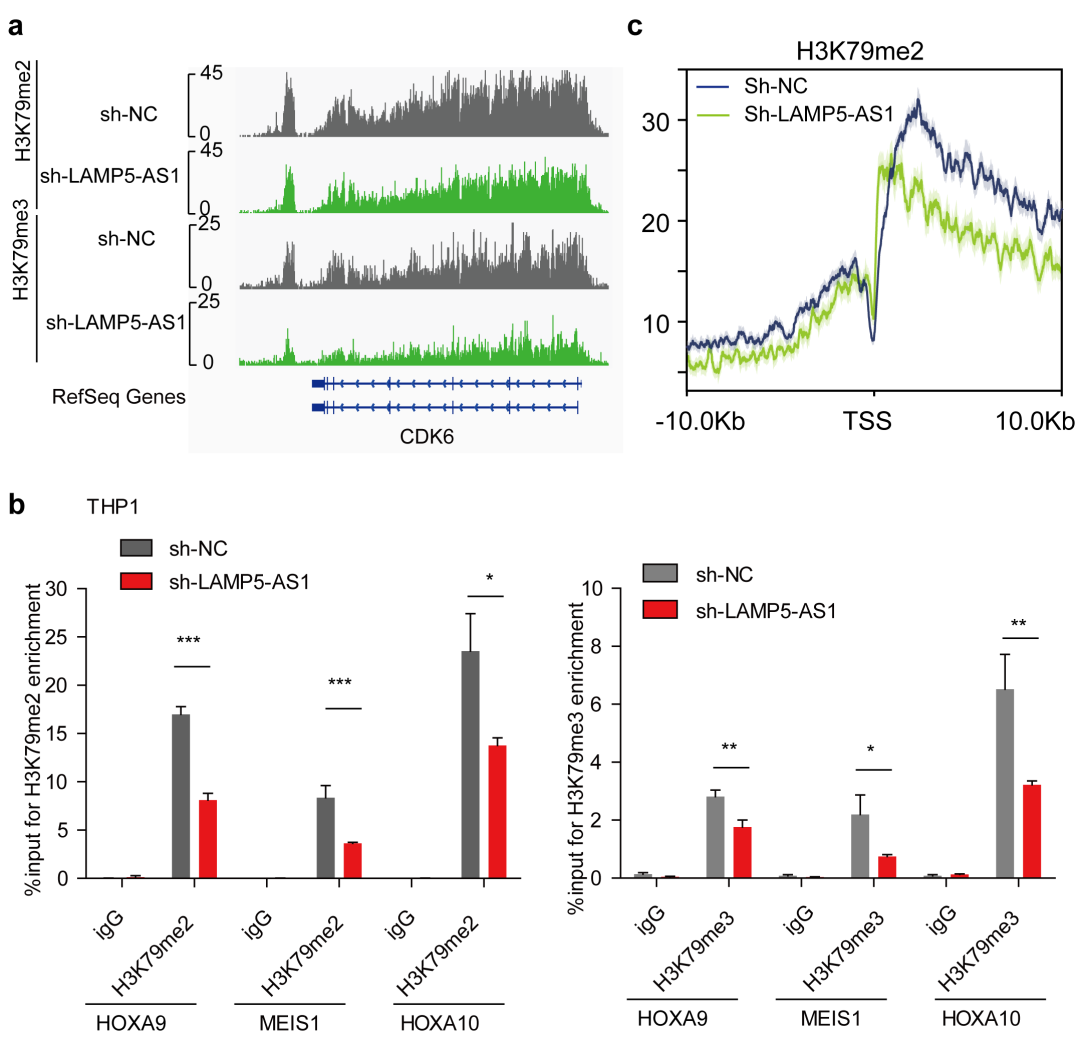


**Figure S5. Epigenomic changes upon LAMP5-AS1 knockdown. a** ChIP-seq profiles of H3K79me2 and H3K79me3 at the *CDK6* genomic loci in LAMP5-AS1-knockdown (green) compared with control (gray) MOLM13 cells. The y-axis scales represent read density per million sequenced reads. **b** H3K79me2(left) and H3K79me3(right) ChIP-qPCR for the core target genes of MLL fusion protein in the LAMP5-AS1 knockdown (red) compared with control (gray) established MOLM13 cells. Error bars reflect ± SEM (*, p<0.05) from three independent experiments. **c** Representative meta-analysis plot showing H3K79me2 profile across the +10 kb to -10 kb genomic region around the TSS of MLL-AF9 target genes. Profiles of LAMP5-AS1-knockdown (green) compared with control (blue) MOLM13 cells are presented.


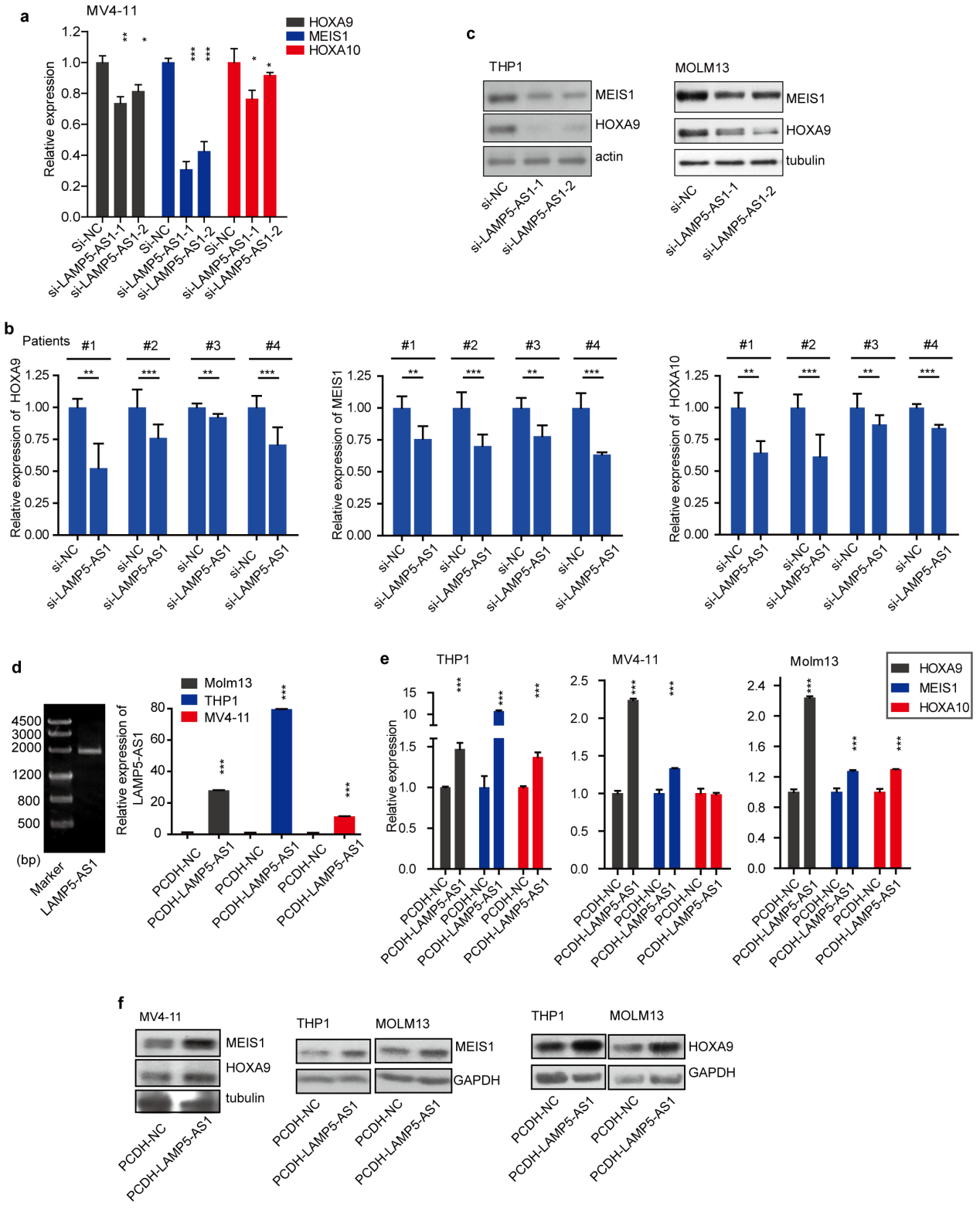


**Figure S6. Genomic changes upon LAMP5-AS1 knockdown or overexpression.** **a** qRT-PCR analysis determined that the expression levels of the MLL fusion protein target genes including *HOXA9, HOXA10* and *MEIS1* were decreased upon LAMP5-AS1 knockdown in MV4-11 cells. Error bars reflect ± SEM (*, p<0.05, **, p<0.01; ***, p<0.001) in three independent experiments. **b** qRT-PCR analysis determined that the expression levels of the MLL fusion protein target genes including *HOXA9, HOXA10* and *MEIS1* were decreased upon LAMP5-AS1 knockdown in 4 primary *MLL* leukemia cells. Error bars reflect ± SEM (*, p<0.05, **, p<0.01; ***, p<0.001) in three independent experiments. **c** Western blotting for the protein levels of HOXA9 and Mesi1 in *MLL* leukemia cells transduced by LAMP5-AS1 siRNA and control. **d** Overexpression of LAMP5-AS1 transcript 1 in *MLL* leukemia cells (MOLM13, MV4-11, and THP1). **e** qRT-PCR analysis determined that the expression levels of the MLL fusion protein target genes including *HOXA9, HOXA10* and *MEIS1* were increased in *MLL* leukemia cell lines treated with LAMP5-AS1 overexpression. Error bars reflect ± SEM (*, p<0.05, **, p<0.01; ***, p<0.001) in three independent experiments. **f** Immunoblot showing the protein levels of HOXA9 and Mesi1 upregulated upon overexpression of LAMP5-AS1 in *MLL* leukemia cell lines.

**Table S1. Patient demographics and clinicopathologic features.**

| Category | *MLL* leukemia (n=58) | *MLL*-wt  leukemia (n=163) | Healthy control (n=5) |
| --- | --- | --- | --- |
| Age at diagnosis(years, Median (range)) | 2.23 (0.16–14) | 5.8(0.83-16) |  |
| WBC count, × 10^9^/L(Median (range)) | 63(2.2-645.84) | 19.5(0.75-557.68) |  |
| Fusion gene |  |  |  |
| *MLL-AF4* | 16 | N/A |  |
| *MLL-AF9* | 7 | N/A |  |
| *MLL-ENL* | 6 | N/A |  |
| *MLL-AF10* | 4 | N/A |  |
| Others | 25 | N/A |  |
| Sex |  |  |  |
| Male | 34 | 107 |  |
| Female | 24 | 56 |  |
| Immunophenotype |  |  |  |
| B | 39 | 87 |  |
| T | 8 | 26 |  |
| AML | 3 | 14 |  |
| Missing | 8 | 36 |  |

**Table S2. Demographics and clinicopathologic features of primary *MLL* leukemia patient samples.**

| Category | Age at diagnosis(years) | WBC count, × 10^9^/L | Fusion gene | Sex | AML/ALL |
| --- | --- | --- | --- | --- | --- |
| Patient #1 | 0.58 | 645.84 | *MLL-AF4* | Male | B-ALL |
| Patient #2 | 1.42 | 160.02 | *MLL-AF9* | Female | B-ALL |
| Patient #3 | 0.17 | 898 | *MLL*（+） | Female | B-ALL |
| Patient #4 | 2 | / | *MLL-AF10* | Male | AML |
| Patient #5 | 5.75 | 20.5 | *MLL-AF9* | Male | B-ALL |

**Table S3. The primers used in this work.**

| primers | sequences（5' → 3'） | |  |
| --- | --- | --- | --- |
| RACE primer | |  | |
| 5’RACE primer-outer-R | | CTGCAGTCTCCAAGGGACAGTGATGCTACTGGTC | |
| 5’RACE primer-outer-F | | CTAATACGACTCACTATAGGGCAAGCAGTGGTATCAACGCAGAGT | |
| 5’RACE primer-Nested-R | | AGCGGCGACTTTGAGGGATT | |
| 5’RACE primer-Nested-F | | CTAATACGACTCACTATAGGGC | |
| 3’RACE primer-outer-R | | GCTGTCAACGATACGCTACGTAACG | |
| 3’RACE primer-outer-F | | CACTGAACGGATCTCAAACC | |
| 3’RACE primer-Nested-R | | CGCTACGTAACGGCATGACAGTG | |
| 3’RACE primer-Nested-F | | TGACAACCTGTTCAACCCTG | |
| qPCR primers | |  | |
| qLAMP5-AS1-transcript 1-Forward | | TTTGCCGGCTCTGTTCTCTG | |
| qLAMP5-AS1-transcript 1-Reverse | | TGTTGTTCCGTGAGTAGCGA | |
| qLAMP5-AS1-transcript 2-Forward | | CAGGGGAACAGAAGGCATTAC | |
| qLAMP5-AS1-transcript 2-Reverse | | GATGTTGTTCCGTGAGTAGCG | |
| qLAMP5-AS1-Forward | | CACTGAACGGATCTCAAACC | |
| qLAMP5-AS1-Reverse | | CCAAGGGACAGTGATGCTAC | |
| qGAPDH-Forward | | GAGTCAACGGATTTGGTCGTAT | |
| qGAPDH-Reverse | | ATGGGTGGAATCATATTGGAAC | |
| qHOXA9-Forward | | GAGGCAGGTCAAGATCTGGTTCC | |
| qHOXA9-Reverse | | GATGTGGCCTGAGGTTTAGAGC | |
| qHOXA10-Forward | | CTCACGGACAGACAAGTGAAAATC | |
| qHOXA10-Reverse | | CAGCCCTGCACAGATGTAACG | |
| qMEIS1-Forward | | TGGGCTTGGCGTTATTATAC | |
| qMEIS1-Reverse | | CAAATGCTTGTTGATAGCCTT | |
| ChIP primers for qPCR | |  | |
| ChIP-HOXA9- Forward | | TGGGCAACTACTACGTGGACTC | |
| ChIP-HOXA9- Reverse | | AAACACCGTCGCCTTGGA | |
| ChIP-HOXA10- Forward | | GGAGGTGGCCGATGTCTAAG | |
| ChIP-HOXA10- Reverse | | CACAGCTGGAACACTTTCGC | |
| ChIP-MEIS1- Forward | | GATGGTACCCAGTTGCTGCT | |
| ChIP-MEIS1- Reverse | | GCCACAGCTCTACCATCTCC | |
| PCR primers | |  | |
| pLAMP5-AS1-1-Forward | | CGGAATTCAAGCTAATTGACTTTACTTTGCC | |
| pLAMP5-AS1-503-Forwad | | TAATACGACTCACTATAGGGACATTCGAGCAGCTGCTCTG | |
| pLAMP5-AS1-601-Forward | | TAATACGACTCACTATAGGGcttgtagcccagagcagca | |
| pLAMP5-AS1-765-Forward | | TAATACGACTCACTATAGGGttggagccagctgcagct | |
| pLAMP5-AS1-502-Reverse | | TGCCAAGGAGCCCAACTCG | |
| pLAMP5-AS1-1036-Reverse | | TTGGAGCGGAGTTGGAGTT | |
| pLAMP5-AS1-764-Reverse | | tggctccaagtgcgtggccggaggcgtcc | |
| pLAMP5-AS1-1928-Reverse | | CGGGATCCGACCTCATAAACTAATATTTATTGAATT | |
| qLAMP5-AS1-AS-Forwad | | TAATACGACTCACTATAGGGGACCTCATAAACTAATATTTATTGAATT | |
| qLAMP5-AS1-AS-Reverse | | aagctaattgactttactttgccggc | |
| ptRSA-LAMP5-AS1-1-Forward | | GACTAGTCTAGAAAGCTAATTGACTTTACTTTGCC | |
| ptRSA-LAMP5-AS1-1-Reverse | | CACACGGAATTCGACCTCATAAACTAATATTTATTGAATT | |
| ptRSA-LAMP5-AS1-502-Reverse | | CACACGGAATTCTGCCAAGGAGCCCAACT | |
| ptRSA-LAMP5-AS1-503-Forward | | GACTAGTCTAGAACATTCGAGCAGCTGCTCTG | |
| ptRSA-LAMP5-AS1-1036-Reverse | | CACACGGAATTCTTGGAGCGGAGTTGGAGT | |
| ptRSA-LAMP5-AS1-1037-Forward | | GACTAGTCTAGAAGAGTGGGAAGCCAGAGG | |
| pDOT1L-Forward | | CGGAATTCATGGGGGAGAAGCTGGAGCT | |
| pDOT1L-Reverse | | CCGCTCGAGGTTACCTCCAACTGTGCCGCCT | |
| pDOT1L-360aa-Forward | | CGGAATTC ATGCCAGAGGGCAAGGTGGC | |
| pDOT1L-390aa-Reverse | | CCGCTCGAGCGGCTTCTTCACGGTGGCTGCT | |
| pDOT1L-1-416aa(det390-407aa)-Reverse | | CCGCTCGAGCTTCTTGGGGCGCCCGCGCTTGCGGCC CGGCTTCTTCACGGTGGCTGCT | |
| pDOT1L-416aa-Reverse | | CCGCTCGAGCTTCTTGGGGCGCCCGCGCT | |
| pDOT1L-417aa-Forward | | CGGAATTC ATGAACACTGCGAACCCC | |
| pDOT1L-822aa-Reverse | | CCGCTCGAGGCTGCCAGGCACGCTGGGGC | |
| pDOT1L-823aa-Forward | | CGGAATTC ATGAAGCTGAGCCCTCAGGA | |

**Table S4.** **siRNA/shRNA.**

| SiRNA/shRNA | sequences（5' → 3'） |
| --- | --- |
| Si-LAMP5-AS1-1-S | 5‘ CUGACAAAGUGCCGUCCAA dTdT 3‘ |
| Si-LAMP5-AS1-1-AS | 3‘ dTdT GACUGUUUCACGGCAGGUU 5‘ |
| Si-LAMP5-AS1-2-S | 5‘ GAGGCAAGACGAAGAAAGU dTdT 3 |
| Si-LAMP5-AS1-2-AS | 3‘ dTdT CUCCGUUCUGCUUCUUUCA 5‘ |
| Sh-LAMP5-AS1-1-S | 5’GATCCCTGACAAAGTGCCGTCCAATTCAAGAGATTGGACGGCACTTTGTCAGTTTTTG3’ |
| Sh-LAMP5-AS1-1-AS | 5’AATTCAAAAACTGACAAAGTGCCGTCCAATCTCTTGAATTGGACGGCACTTTGTCAGG3’ |
| Sh-LAMP5-AS1-2-S | 5’GATCCGAGGCAAGACGAAGAAAGTTTCAAGAGAACTTTCTTCGTCTTGCCACTTTTTG3’ |
| Sh-LAMP5-AS1-2-AS | 5’AATTCAAAAAGAGGCAAGACGAAGAAAGTTCTCTTGAAACTTTCTTCGTCTTGCCTCG3’ |

**Table S5. ALL of the antibodies and regents used in this study.**

| namer | species | company | NO. | Storage |
| --- | --- | --- | --- | --- |
| HA-Tag (C29F4) mAb | Rabbit | CST | 3724 | -20℃ |
| Anti-HA mAb | Mouse | Sigma | H9658 | -20℃ |
| FLAG | Rabbit | Sigma | F7425 | -20℃ |
| FLAG | Mouse | Abmart | M20008M | -20 |
| β-tubulin | Mouse | invitrogen | 32-2600 | -20℃ |
| GAPDH polyAb | Rabbit | Proteintech | 10494-1-AP | -20℃ |
| β-actin | Mouse | Sigma | A2228 | -20℃ |
| DOT1L1 (H-300) pAb | Rabbit | Santa Cruz | sc-292233 | 4℃ |
| DOT1L (D4O2T) | Rabbit | CST | 90878S | -20 |
| Histone H3 (tri methyl K79)- ChIP Grade | Rabbit | Abcam | ab2621 | -20 |
| Histone H3 (di methyl K79)- ChIP Grade  (tri methyl K79)- ChIP Grade | Rabbit | Abcam | ab3594 | -20 |
| Histone H3 | Rabbit | Abcam | Ab1791 | -20℃ |
| lamin A/C | Mouse | CST | 4777 | -20℃ |
| HOXA9 | Rabbit | Abcam | ab140631 | -20℃ |
| MEIS1 | Rabbit | Abcam | ab19867 | -20℃ |
| MLL1 | Rabbit | BETHYL | A300-086A | 4℃ |
| Goat Anti-Mouse IgG HRP | Mouse | Thermo Fisher | H10007 | -20℃ |
| Goat Anti-Rabbit IgG HRP | Rabbit | Thermo Fisher | A18903 | -20℃ |
| Goat Anti-Rabbit IgG(H+L) AF488 | Rabbit | Transgene | K21017 | 4℃ |
| CD14 PE mouse anti-human | Human | eBioscience | 12-0149 | 4℃ |
| CD11b/Mac-1 APC mouse anti-Human | Human | BD | 9196064 | 4℃ |
| CD34 Monoclonal Antibody (4H11), FITC | Human | eBioscience | 11-0349-42 | 4℃ |
| APC Mouse Anti-Human CD19 Clone HIB19 | Human | BD | 561742 | 4℃ |
| Ribo^TM^ LAMP5-AS1 FISH Probe Mix(Cy3) | Human | Ribobio | 101929329 | -20℃ |
| Human native nucleosomes | Human | Milliproe | 14-1057 | -80℃ |
| IL-3 | Human | PeproTech | 200-03 | -80℃ |
| IL-6 | Human | PeproTech | 200-06 | -80℃ |
| SCF | Human | PeproTech | 300-07 | -80℃ |
| Flt3-Ligand | Human | PeproTech | 300-19 | -80℃ |
| TPO | Human | PeproTech | 300-18 | -80℃ |
| GM-CSF | Human | PeproTech | 300-03 | -80℃ |
| Thrombin | - | Sigma | T4648-1KU | -20℃ |

**Table S6. MS of proteins from LAMP5-AS1 pull down.**

| Accession | Name | #Peptides | #Unique | Description |
| --- | --- | --- | --- | --- |
| Q9P2E9 | RRBP1 | 35 | 34 | Ribosome-binding protein 1 OS=Homo sapiens GN=RRBP1 PE=1 SV=4 |
| Q6P2Q9 | PRPF8 | 35 | 34 | Pre-mRNA-processing-splicing factor 8 OS=Homo sapiens GN=PRPF8 PE=1 SV=2 |
| P46940 | IQGAP1 | 33 | 33 | Ras GTPase-activating-like protein IQGAP1 OS=Homo sapiens GN=IQGAP1 PE=1 SV=1 |
| P35579 | MYH9 | 39 | 32 | Myosin-9 OS=Homo sapiens GN=MYH9 PE=1 SV=4 |
| Q14008 | CKAP5 | 31 | 31 | Cytoskeleton-associated protein 5 OS=Homo sapiens GN=CKAP5 PE=1 SV=3 |
| O75643 | SNRNP200 | 31 | 31 | U5 small nuclear ribonucleoprotein 200 kDa helicase OS=Homo sapiens GN=SNRNP200 PE=1 SV=2 |
| Q8TEK3 | DOT1L | 23 | 23 | Histone-lysine N-methyltransferase H3 lysine-79 specific OS=Homo sapiens GN=DOT1L PE=1 SV=2 |
| Q13686 | ALKBH1 | 20 | 20 | Nucleic acid dioxygenase ALKBH1 OS=Homo sapiens GN=ALKBH1 PE=1 SV=2 |
| Q14152 | EIF3A | 16 | 16 | Eukaryotic translation initiation factor 3 subunit A OS=Homo sapiens GN=EIF3A PE=1 SV=1 |
| Q92608 | DOCK2 | 16 | 16 | Dedicator of cytokinesis protein 2 OS=Homo sapiens GN=DOCK2 PE=1 SV=2 |
| P35580 | MYH10 | 24 | 15 | Myosin-10 OS=Homo sapiens GN=MYH10 PE=1 SV=3 |
| P02768 | ALB | 21 | 14 | Serum albumin OS=Homo sapiens GN=ALB PE=1 SV=2 |
| P27816 | MAP4 | 14 | 14 | Microtubule-associated protein 4 OS=Homo sapiens GN=MAP4 PE=1 SV=3 |
| Q04637 | EIF4G1 | 12 | 12 | Eukaryotic translation initiation factor 4 gamma 1 OS=Homo sapiens GN=EIF4G1 PE=1 SV=4 |
| Q9C0B1 | FTO | 12 | 12 | Alpha-ketoglutarate-dependent dioxygenase FTO OS=Homo sapiens GN=FTO PE=1 SV=3 |
| P48634 | PRRC2A | 10 | 10 | Protein PRRC2A OS=Homo sapiens GN=PRRC2A PE=1 SV=3 |
| Q8N3C0 | ASCC3 | 10 | 10 | Activating signal cointegrator 1 complex subunit 3 OS=Homo sapiens GN=ASCC3 PE=1 SV=3 |
| P07814 | EPRS | 10 | 10 | Bifunctional glutamate/proline--tRNA ligase OS=Homo sapiens GN=EPRS PE=1 SV=5 |
| Q10570 | CPSF1 | 9 | 9 | Cleavage and polyadenylation specificity factor subunit 1 OS=Homo sapiens GN=CPSF1 PE=1 SV=2 |
| O60841 | EIF5B | 8 | 8 | Eukaryotic translation initiation factor 5B OS=Homo sapiens GN=EIF5B PE=1 SV=4 |
| P26358 | DNMT1 | 8 | 8 | DNA (cytosine-5)-methyltransferase 1 OS=Homo sapiens GN=DNMT1 PE=1 SV=2 |
| Q9NTJ3 | SMC4 | 8 | 8 | Structural maintenance of chromosomes protein 4 OS=Homo sapiens GN=SMC4 PE=1 SV=2 |
| P04264 | KRT1 | 13 | 7 | Keratin type II cytoskeletal 1 OS=Homo sapiens GN=KRT1 PE=1 SV=6 |
| P13645 | KRT10 | 8 | 7 | Keratin type I cytoskeletal 10 OS=Homo sapiens GN=KRT10 PE=1 SV=6 |
| Q08211 | DHX9 | 7 | 7 | ATP-dependent RNA helicase A OS=Homo sapiens GN=DHX9 PE=1 SV=4 |
| P24928 | POLR2A | 7 | 7 | DNA-directed RNA polymerase II subunit RPB1 OS=Homo sapiens GN=POLR2A PE=1 SV=2 |
| O95248 | SBF1 | 6 | 6 | Myotubularin-related protein 5 OS=Homo sapiens GN=SBF1 PE=1 SV=3 |
| O94813 | SLIT2 | 6 | 6 | Slit homolog 2 protein OS=Homo sapiens GN=SLIT2 PE=1 SV=1 |
| Q9HAU5 | UPF2 | 6 | 6 | Regulator of nonsense transcripts 2 OS=Homo sapiens GN=UPF2 PE=1 SV=1 |
| Q8N201 | INTS1 | 6 | 6 | Integrator complex subunit 1 OS=Homo sapiens GN=INTS1 PE=1 SV=2 |
| Q8TEQ6 | GEMIN5 | 6 | 6 | Gem-associated protein 5 OS=Homo sapiens GN=GEMIN5 PE=1 SV=3 |
| O75165 | DNAJC13 | 6 | 6 | DnaJ homolog subfamily C member 13 OS=Homo sapiens GN=DNAJC13 PE=1 SV=5 |
| P35527 | KRT9 | 5 | 5 | Keratin type I cytoskeletal 9 OS=Homo sapiens GN=KRT9 PE=1 SV=3 |
| Q14669 | TRIP12 | 5 | 5 | E3 ubiquitin-protein ligase TRIP12 OS=Homo sapiens GN=TRIP12 PE=1 SV=1 |
| Q00839 | HNRNPU | 4 | 4 | Heterogeneous nuclear ribonucleoprotein U OS=Homo sapiens GN=HNRNPU PE=1 SV=6 |
| Q8IZH2 | XRN1 | 4 | 4 | 5'-3' exoribonuclease 1 OS=Homo sapiens GN=XRN1 PE=1 SV=1 |
| P19338 | NCL | 4 | 4 | Nucleolin OS=Homo sapiens GN=NCL PE=1 SV=3 |
| O94913 | PCF11 | 4 | 4 | Pre-mRNA cleavage complex 2 protein Pcf11 OS=Homo sapiens GN=PCF11 PE=1 SV=3 |
| P01859 | IGHG2 | 4 | 4 | Immunoglobulin heavy constant gamma 2 OS=Homo sapiens GN=IGHG2 PE=1 SV=2 |
| P35908 | KRT2 | 7 | 3 | Keratin type II cytoskeletal 2 epidermal OS=Homo sapiens GN=KRT2 PE=1 SV=2 |
| Q69YN4 | KIAA1429 | 3 | 3 | Protein virilizer homolog OS=Homo sapiens GN=KIAA1429 PE=1 SV=2 |
| Q8NI27 | THOC2 | 3 | 3 | THO complex subunit 2 OS=Homo sapiens GN=THOC2 PE=1 SV=2 |
| Q6Y7W6 | GIGYF2 | 3 | 3 | GRB10-interacting GYF protein 2 OS=Homo sapiens GN=GIGYF2 PE=1 SV=1 |
| Q96N67 | DOCK7 | 3 | 3 | Dedicator of cytokinesis protein 7 OS=Homo sapiens GN=DOCK7 PE=1 SV=4 |
| P01011 | SERPINA3 | 2 | 2 | Alpha-1-antichymotrypsin OS=Homo sapiens GN=SERPINA3 PE=1 SV=2 |
| Q9NYV4 | CDK12 | 2 | 2 | Cyclin-dependent kinase 12 OS=Homo sapiens GN=CDK12 PE=1 SV=2 |
| P55196 | AFDN | 2 | 2 | Afadin OS=Homo sapiens GN=AFDN PE=1 SV=3 |
| P68104 | EEF1A1 | 2 | 2 | Elongation factor 1-alpha 1 OS=Homo sapiens GN=EEF1A1 PE=1 SV=1 |
| Q5VTE0 | EEF1A1P5 | 2 | 2 | Putative elongation factor 1-alpha-like 3 OS=Homo sapiens GN=EEF1A1P5 PE=5 SV=1 |
| Q05639 | EEF1A2 | 2 | 2 | Elongation factor 1-alpha 2 OS=Homo sapiens GN=EEF1A2 PE=1 SV=1 |
| P00450 | CP | 2 | 2 | Ceruloplasmin OS=Homo sapiens GN=CP PE=1 SV=1 |
| P02790 | HPX | 2 | 2 | Hemopexin OS=Homo sapiens GN=HPX PE=1 SV=2 |
| Q13428 | TCOF1 | 2 | 2 | Treacle protein OS=Homo sapiens GN=TCOF1 PE=1 SV=3 |
| P78527 | PRKDC | 3 | 2 | DNA-dependent protein kinase catalytic subunit OS=Homo sapiens GN=PRKDC PE=1 SV=3 |
| P42695 | NCAPD3 | 3 | 2 | Condensin-2 complex subunit D3 OS=Homo sapiens GN=NCAPD3 PE=1 SV=2 |
| P62987 | UBA52 | 2 | 2 | Ubiquitin-60S ribosomal protein L40 OS=Homo sapiens GN=UBA52 PE=1 SV=2 |
| P62979 | RPS27A | 2 | 2 | Ubiquitin-40S ribosomal protein S27a OS=Homo sapiens GN=RPS27A PE=1 SV=2 |
| P0CG47 | UBB | 2 | 2 | Polyubiquitin-B OS=Homo sapiens GN=UBB PE=1 SV=1 |
| P0CG48 | UBC | 2 | 2 | Polyubiquitin-C OS=Homo sapiens GN=UBC PE=1 SV=3 |
| O14578 | CIT | 2 | 2 | Citron Rho-interacting kinase OS=Homo sapiens GN=CIT PE=1 SV=2 |
| Q8N3X1 | FNBP4 | 2 | 2 | Formin-binding protein 4 OS=Homo sapiens GN=FNBP4 PE=1 SV=3 |
| Q14692 | BMS1 | 2 | 2 | Ribosome biogenesis protein BMS1 homolog OS=Homo sapiens GN=BMS1 PE=1 SV=1 |
| Q5JSL3 | DOCK11 | 1 | 1 | Dedicator of cytokinesis protein 11 OS=Homo sapiens GN=DOCK11 PE=1 SV=2 |
| Q9Y520 | PRRC2C | 1 | 1 | Protein PRRC2C OS=Homo sapiens GN=PRRC2C PE=1 SV=4 |
| P02774 | GC | 1 | 1 | Vitamin D-binding protein OS=Homo sapiens GN=GC PE=1 SV=1 |
| Q8IWZ3 | ANKHD1 | 2 | 1 | Ankyrin repeat and KH domain-containing protein 1 OS=Homo sapiens GN=ANKHD1 PE=1 SV=1 |
| P02533 | KRT14 | 2 | 1 | Keratin type I cytoskeletal 14 OS=Homo sapiens GN=KRT14 PE=1 SV=4 |
| P08727 | KRT19 | 2 | 1 | Keratin type I cytoskeletal 19 OS=Homo sapiens GN=KRT19 PE=1 SV=4 |
| Q04695 | KRT17 | 2 | 1 | Keratin type I cytoskeletal 17 OS=Homo sapiens GN=KRT17 PE=1 SV=2 |
| P19012 | KRT15 | 2 | 1 | Keratin type I cytoskeletal 15 OS=Homo sapiens GN=KRT15 PE=1 SV=3 |
| P08779 | KRT16 | 2 | 1 | Keratin type I cytoskeletal 16 OS=Homo sapiens GN=KRT16 PE=1 SV=4 |
| Q96EV2 | RBM33 | 1 | 1 | RNA-binding protein 33 OS=Homo sapiens GN=RBM33 PE=1 SV=3 |
| P61626 | LYZ | 1 | 1 | Lysozyme C OS=Homo sapiens GN=LYZ PE=1 SV=1 |
| P51531 | SMARCA2 | 1 | 1 | Probable global transcription activator SNF2L2 OS=Homo sapiens GN=SMARCA2 PE=1 SV=2 |
| P51532 | SMARCA4 | 1 | 1 | Transcription activator BRG1 OS=Homo sapiens GN=SMARCA4 PE=1 SV=2 |
| Q9Y5S2 | CDC42BPB | 1 | 1 | Serine/threonine-protein kinase MRCK beta OS=Homo sapiens GN=CDC42BPB PE=1 SV=2 |
| O75179 | ANKRD17 | 2 | 1 | Ankyrin repeat domain-containing protein 17 OS=Homo sapiens GN=ANKRD17 PE=1 SV=3 |
| P62861 | FAU | 1 | 1 | 40S ribosomal protein S30 OS=Homo sapiens GN=FAU PE=1 SV=1 |
| P16403 | HIST1H1C | 1 | 1 | Histone H1.2 OS=Homo sapiens GN=HIST1H1C PE=1 SV=2 |
| P16402 | HIST1H1D | 1 | 1 | Histone H1.3 OS=Homo sapiens GN=HIST1H1D PE=1 SV=2 |
| P10412 | HIST1H1E | 1 | 1 | Histone H1.4 OS=Homo sapiens GN=HIST1H1E PE=1 SV=2 |
| Q8IY21 | DDX60 | 1 | 1 | Probable ATP-dependent RNA helicase DDX60 OS=Homo sapiens GN=DDX60 PE=1 SV=3 |
| P81605 | DCD | 1 | 1 | Dermcidin OS=Homo sapiens GN=DCD PE=1 SV=2 |
| Q14980 | NUMA1 | 1 | 1 | Nuclear mitotic apparatus protein 1 OS=Homo sapiens GN=NUMA1 PE=1 SV=2 |
| P07477 | PRSS1 | 2 | 1 | Trypsin-1 OS=Homo sapiens GN=PRSS1 PE=1 SV=1 |
| A6NHR9 | SMCHD1 | 1 | 1 | Structural maintenance of chromosomes flexible hinge domain-containing protein 1 OS=Homo sapiens GN=SMCHD1 PE=1 SV=2 |
| P0C0L5 | C4B | 1 | 1 | Complement C4-B OS=Homo sapiens GN=C4B PE=1 SV=2 |
| P0C0L4 | C4A | 1 | 1 | Complement C4-A OS=Homo sapiens GN=C4A PE=1 SV=2 |
| P39060 | COL18A1 | 1 | 1 | Collagen alpha-1(XVIII) chain OS=Homo sapiens GN=COL18A1 PE=1 SV=5 |
| P04114 | APOB | 1 | 1 | Apolipoprotein B-100 OS=Homo sapiens GN=APOB PE=1 SV=2 |
| P02753 | RBP4 | 1 | 1 | Retinol-binding protein 4 OS=Homo sapiens GN=RBP4 PE=1 SV=3 |
| Q86US8 | SMG6 | 1 | 1 | Telomerase-binding protein EST1A OS=Homo sapiens GN=SMG6 PE=1 SV=2 |
| Q5THJ4 | VPS13D | 1 | 1 | Vacuolar protein sorting-associated protein 13D OS=Homo sapiens GN=VPS13D PE=1 SV=2 |
| O95602 | POLR1A | 1 | 1 | DNA-directed RNA polymerase I subunit RPA1 OS=Homo sapiens GN=POLR1A PE=1 SV=2 |
| Q14690 | PDCD11 | 1 | 1 | Protein RRP5 homolog OS=Homo sapiens GN=PDCD11 PE=1 SV=3 |
| Q05707 | COL14A1 | 1 | 1 | Collagen alpha-1(XIV) chain OS=Homo sapiens GN=COL14A1 PE=1 SV=3 |
| Q92572 | AP3S1 | 1 | 1 | AP-3 complex subunit sigma-1 OS=Homo sapiens GN=AP3S1 PE=1 SV=1 |
| Q96CB9 | NSUN4 | 1 | 1 | 5-methylcytosine rRNA methyltransferase NSUN4 OS=Homo sapiens GN=NSUN4 PE=1 SV=2 |
| Q99946 | PRRT1 | 1 | 1 | Proline-rich transmembrane protein 1 OS=Homo sapiens GN=PRRT1 PE=2 SV=2 |
| P08195 | SLC3A2 | 1 | 1 | 4F2 cell-surface antigen heavy chain OS=Homo sapiens GN=SLC3A2 PE=1 SV=3 |
| P51530 | DNA2 | 1 | 1 | DNA replication ATP-dependent helicase/nuclease DNA2 OS=Homo sapiens GN=DNA2 PE=1 SV=3 |
